# Metabolic pathway and cell adaptation mechanisms revealed through genomic, proteomic and transcription analysis of a *Sphingomonas haloaromaticamans* strain degrading *ortho*-phenylphenol

**DOI:** 10.1038/s41598-017-06727-6

**Published:** 2017-07-25

**Authors:** Chiara Perruchon, Sotirios Vasileiadis, Constantina Rousidou, Evangelia S. Papadopoulou, Georgia Tanou, Martina Samiotaki, Constantinos Garagounis, Athanasios Molassiotis, Kalliope K. Papadopoulou, Dimitrios G. Karpouzas

**Affiliations:** 1 Department of Biochemistry and Biotechnology, University of Thessaly, Laboratory of Plant and Environmental Biotechnology, Viopolis, 41500 Larissa Greece; 2 University of South Australia, Future Industries Institute, Mawson Lakes, Australia; 3 Aristotle University of Thessaloniki, School of Agriculture, Thessaloniki, Greece; 4 Biomedical Sciences Research Center “Alexander Fleming”, Vari, 16672 Greece

## Abstract

*Ortho*-phenylphenol (OPP) is a fungicide contained in agro-industrial effluents produced by fruit-packaging plants. Within the frame of developing bio-strategies to detoxify these effluents, an OPP-degrading *Sphingomonas haloaromaticamans* strain was isolated. Proteins/genes with a putative catabolic role and bacterium adaptation mechanisms during OPP degradation were identified via genomic and proteomic analysis. Transcription analysis of all putative catabolic genes established their role in the metabolism of OPP. The formation of key transformation products was verified by chromatographic analysis. Genomic analysis identified two orthologous operons encoding the *ortho*-cleavage of benzoic acid (BA) (*ben/cat*). The second *ben/cat* operon was located in a 92-kb scaffold along with (i) an operon (*opp*) comprising genes for the transformation of OPP to BA and 2-hydroxypenta-2,4-dienoate (and genes for its transformation) and (ii) an incomplete biphenyl catabolic operon (*bph*). Proteomics identified 13 up-regulated catabolic proteins when *S. haloaromaticamans* was growing on OPP and/or BA. Transcription analysis verified the key role of the catabolic operons located in the 92-kb scaffold, and flanked by transposases, on the transformation of OPP by *S. haloaromaticamans*. A flavin-dependent monoxygenase (OppA1), one of the most up-regulated proteins in the OPP-growing cells, was isolated via heterologous expression and its catabolic activity was verified *in vitro*.

## Introduction

*Ortho*-phenylphenol (OPP) is used in the post-harvest treatment of fruits to control fungal infestations during storage^1^. Its application results in the production of large wastewater volumes which require treatment on site^2^. The development of biological treatment systems based on the specific ability of microorganisms to degrade OPP could be a viable solution for the detoxification of these effluents. In this context, a *Sphingomonas haloaromaticamans* strain P3 was recently isolated from a soil collected from a wastewater disposal site^3^. The bacterium is using OPP as a carbon source and showed high potential for application in biodepuration and bioaugmentation strategies. However, the microbial metabolic pathway of the fungicide and the genetic systems driving its degradation by strain P3 remain unknown. Biodegradation is not always synonymous to detoxification; instead it occasionally leads to the formation of metabolic products that are more toxic or persistent than the parent compound^4^. Therefore, elucidation of the microbial metabolic pathway of OPP is a prerequisite for the downstream exploitation of strain P3 in any environmental application.

To date little is known about the microbial degradation of OPP. Kohler *et al*.^5^ were the first, and the only other study to date, that have studied the microbial degradation of OPP. They isolated a *Pseudomonas azelaica* strain HPB1 which was able to degrade OPP through the production of 2,3-dihydroxybiphenyl. Jaspers *et al*.^6^ identified a gene cluster, *hbpCAD*, encoding the upper metabolic pathway of OPP which involves the transformation of OPP to 2-hydroxypenta-2,4-dienoateand benzoic acid (BA). The downstream transformation of BA involved a *meta*-cleavage pathway, although its genetic organization and function was not revealed and the overall network of genes driving the full metabolic pathway of OPP is still not known. HbpA, a flavin-dependent monoxygenase responsible for the initial hydroxylation of OPP by *P. azelaica* HPB1, was isolated^7^ and characterized^8^.

Advances in high-throughput sequencing have shed light into the full genetic armoury of xenobiotic-degrading bacteria^9,10^, making protein annotation, metabolic pathway prediction and reconstruction feasible^11,12^. However, the mere presence of putative catabolic genes in a bacterial genome does not guarantee biodegradability in a given environment^13^. Proteomic analysis offers a unique dynamic view of the catabolic network of xenobiotic-degrading bacteria within the context of the overall cell response and adaptation to pollutant exposure^14–16^. Good knowledge of the physiological response of bacteria during exposure and degradation of organic pollutants is necessary for their future industrial exploitation.

The aim of the present study was to unravel the genetic mechanisms driving the metabolic pathway of OPP and the overall cellular response of *S. haloaromaticamans* strain P3 during degradation of OPP. To achieve this, a combination of genomics and proteomics coupled with transcription and chromatographic analysis was employed. The total bacterial genome was sequenced, assembled, annotated and used for mapping of the proteome of strain P3 growing on OPP, BA and succinate. Up-regulated enzymes with a putative role in the transformation of OPP were identified and their expression patterns during degradation of OPP and BA was determined by reverse transcription (RT)-q-PCR. The key enzyme involved in the first step of the metabolic pathway, a flavin-dependent monoxygenase, was isolated via heterologous expression, purified and its activity against OPP was verified *in vitro*. The combinatory use of advanced omic tools is expected to unravel the full genetic network driving microbial degradation of OPP for the first time as part of the wider cellular response of the studied bacterium to fungicide exposure. This knowledge will facilitate the exploitation of *S. haloaromaticamans* strain P3 in future bioremediation and biodepuration strategies.

## Results and Discussion

### Genomic analysis of *S. haloaromaticamans*

The draft genome of *S. haloaromaticamans* strain P3 had a size of 4.812.401 bp with a mean GC content of 62%, which falls within the average size and GC content values of known sphingomonad genomes (3.4–5.9 Mbp)^17^. Variation in the size and the GC content of the genomes of Sphingomonads has been attributed to the presence of plasmids and genomic islands^18^. A total of 4630 ORFs were predicted. The annotation of the sequenced genome generated 57 large contigs assembled into 13 scaffolds with the main ones being scaffolds 1 (4.5 Mbp), 2 (192 kbp) and 3 (92 kbp).

**Figure 1:**
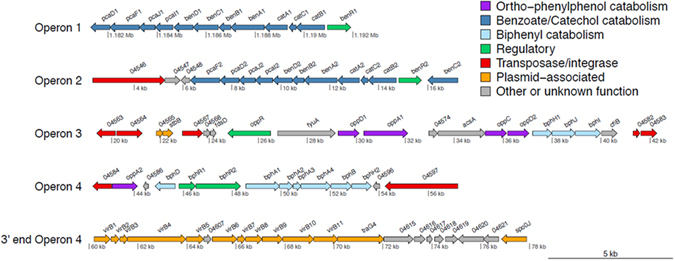
Genetic organization of operons 1, 2, 3 and 4 containing genes involved in the transformation of OPP by *Sphingomonas haloaromaticamans* strain P3. Operon 1 is located in scaffold 1 which represents the bacterial chromosome (4.5 Mb) and encodes a complete *ortho* - cleavage pathway for benzoic acid (BA). Operons 2, 3 and 4 are located in the 92-kb scaffold 3 and encode (2) a second complete *ortho* - cleavage pathway for BA, (3) enzymes with a putative role in the upper pathway of OPP, and a lower biphenyl (*bph*) pathway, and (4) an incomplete upper *bph* pathway. The genomic region downstream of the 3′ end of operon 4 encoding genes of the type IV secretion system and other plasmid conjugation proteins is also shown. Annotation of the different ORFs is shown in Table 1.

Genes with a putative role in the metabolism of aromatic compounds like OPP were localized in four well-organized operons. This is in contrast to other sphingomonads where the genes for the individual degrading pathways are localized in several gene clusters scattered in the genome^19,20^. Operons 1 and 2 encoded a BA *ortho* cleavage pathway (*ben/cat* operon). Operon 3 comprised genes with a putative role in the upper part of the metabolic pathway of OPP (*opp* operon) and genes from the lower biphenyl (*bph*) pathway. Finally operon 4 encoded an incomplete *bph* pathway (Table 1, Fig. 1). From these, only operon 1 was localized in the 4.5-Mb-scaffold, which based on its size, gene organization and the presence of several housekeeping genes could be considered as the bacterial chromosome. The other three catabolic operons were all located in the 92-kb-scaffold 3 and they were separated by transposases and integrases suggesting their acquisition through horizontal gene transfer. Prophages, transposons and insertion elements are common features of sphingomonads and have been deemed responsible for the genome evolution and the high catabolic versatility of this taxon^17,21^. The presence of plasmid stabilization proteins (*stb*) at the 5′ end of operon 3, a near complete Type IV secretion system (virD4 is missing) homologous to the VirB/virD4 secretion system of *Agrobacterium tumefaciens*^22,23^, a conjugal transfer protein (TraG)^24^ and a chromosome partitioning protein (Spo0J) downstream of operon 4^25^ (Fig. 1) strongly suggest that the 55-kb fragment containing operons 2, 3 and 4 is most probably a modular transposon localized in a self-transmissible conjugative plasmid. Recently, Yan *et al*.^10^ showed via comparative genomics the key role of plasmid-localized transposons in the evolution of novel metabolic pathways for the degradation of phenylurea herbicides by Sphingomonads.

**Table 1:** A list of the catabolic genes contained in operons 1, 2, 3 and 4 found in the genome of *S. haloaromaticamans* strain P3. The organization of these genes in the respective operons are presented in Fig. 1.

No.	Locus tag	Gene	Closest homologous protein	Assigned Function
Operon 1 (*ben/cat ortho* cleavage pathway)				
1	BHE75_01127	*pcaD1*	3-oxoadipate enol-lactonase	3-oxoadipate enol-lactonase
2	BHE75_01128	*pcaF1*	Beta-ketoadipyl-CoA thiolase	Beta-ketoadipyl-CoA thiolase
3	BHE75_01129	*pcaJ1*	Succinyl-CoA:3-ketoacid CoA transferase subunit B	Succinyl-CoA:3-ketoacid coenzyme A transferase subunit B
4	BHE75_01130	*pcaI1*	Succinyl-CoA:3-ketoacid CoA transferase subunit A	Succinyl-CoA:3-ketoacid coenzyme A transferase subunit A
5	BHE75_01131	*benD1*	Glucose 1-dehydrogenase B	1,6-dihydroxycyclohexa-2,4-diene-1-carboxylate dehydrogenase
6	BHE75_01132	*benC1*	Benzoate 1,2-dioxygenase electron transfer component	Benzoate 1,2-dioxygenase electron transfer component
7	BHE75_01133	*benB1*	2-halobenzoate 1,2-dioxygenase small subunit	Benzoate 1,2-dioxygenase small subunit
8	BHE75_01134	*benA1*	2-halobenzoate 1,2-dioxygenase large subunit	Benzoate 1,2-dioxygenase large subunit
9	BHE75_01135	*catA1*	Catechol 1-2-dioxygenase	Catechol 1,2-dioxygenase
10	BHE75_01136	*catC1*	Muconolactone Delta-isomerase	Muconolactone δ-isomerase
11	BHE75_01137	*catB1*	Muconate cycloisomerase	Muconate cyclosisomerase
12	BHE75_01138	*benR1*	LysR-type transcriptional regulatory protein	Transcriptional regulatory protein
Operon 2 (*ben/cat ortho* cleavage pathway)				
13	BHE75_04546		Tn3 transposase DDE domain protein	
14	BHE75_04547		Tannase and feruloyl esterase	
15	BHE75_04548		Hypothetical protein	
16	BHE75_04549	*pcaF2*	Beta-ketoadipyl-CoA thiolase	Beta-ketoadipyl-CoA thiolase
17	BHE75_04550	*pcaD2*	3-oxoadipate enol-lactonase	3-oxoadipate enol-lactonase
18	BHE75_04551	*pcaJ2*	Succinyl-CoA:3-ketoacid CoA transferase subunit B	Succinyl-CoA:3-ketoacid CoA transferase subunit B
19	BHE75_04552	*pcaI2*	Succinyl-CoA:3-ketoacid CoA transferase subunit A	Succinyl-CoA:3-ketoacid CoA transferase subunit A
20	BHE75_04553	*benD2*	Levodione reductase	1,6-dihydroxycyclohexa-2,4-diene-1-carboxylate dehydrogenase
21	BHE75_04554	*benB2*	2-halobenzoate 1,2-dioxygenase small subunit	Benzoate 1,2-dioxygenase small subunit
22	BHE75_04555	*benA2*	2-halobenzoate 1,2-dioxygenase large subunit	Benzoate 1,2-dioxygenase large subunit
23	BHE75_04556	*catA2*	Catechol 1,2-dioxygenase	Catechol 1,2-dioxygenase
24	BHE75_04557	*catC2*	Muconolactone δ-isomerase	Muconolactone δ-isomerase
25	BHE75_04558	*catB2*	Muconate cycloisomerase	Muconate cycloisomerase
26	BHE75_04559	*benR2*	HTH-type transcriptional regulator	Transcriptional regulatory protein
27	BHE75_04560	*fdr2/benC2*	Ferredoxin-NAD(P) (+) reductase (putative benzoate 1,2-dioxygenase electron transfer component)	unknown
Operon 3 (upper *opp* pathway & lower *bph* pathway)				
28	BHE75_04563		Integrase core domain protein	
29	BHE75_04564		Integrase	
30	BHE75_04565		Putative plasmid stability protein	
31	BHE75_04566	*stbB*	Putative plasmid stabilization protein	
32	BHE75_04567		Putative integrase	
33	BHE75_04568	*fdsA*	Formate dehydrogenase alpha subunit	
34	BHE75_04569	*fdsD*	NADH-dependant formate dehydrogenase delta subunit	
35	BHE75_04570	*oppR*	XylR_N-type σ^54^-dependent transcriptional regulator	Transcriptional regulatory protein
36	BHE75_04571	*fyuA*	Pesticin receptor precursor	
37	BHE75_04572	*oppD1*	2-hydroxy-6-oxononadienedioate/2-hydroxy-6-oxononatrienedioate hydrolase	2-hydroxy-6-oxo-6-(2′-aminophenyl)hexa-2,4-dienoic acid hydrolase (*or meta*- ring fusion product hydrolase)
38	BHE75_04573	*oppA1*	2,4-dichlorophenol 6-monooxygenase	Flavin-dependent OPP monoxygenase
39	BHE75_04574		Hypothetical protein	
40	BHE75_04575	*acsA*	Acetyl-coenzyme A synthetase	
41	BHE75_04576	*oppC*	3-methylcatechol 2,3-dioxygenase	2,3 dihydroxy, 1,2-dioxygenase
42	BHE75_04577	*oppD2*	2-hydroxymuconate semialdehyde hydrolase	2-hydroxy-6-oxo-6-(2′-aminophenyl)hexa-2,4-dienoic acid hydrolase (or *meta*- ring fusion product hydrolase)
43	BHE75_04578	*bphH1*	2-keto-4-pentenoate hydratase	2-keto-4-pentenoate hydratase
44	BHE75_04579	*bphJ*	Acetaldehyde dehydrogenase	Acetaldehyde dehydrogenase
45	BHE75_04580	*bphI*	4-hydroxy-2-oxovalerate aldolase	4-hydroxy-2-oxovalerate aldolase
46	BHE75_04581	*cfiB*	2-oxoglutarate carboxylase small subunit	
47	BHE75_04582		Transposase	
48	BHE75_04583		Integrase core domain protein	
Operon 4 (upper *bph* pathway)				
49	BHE75_04584		Integrase core domain protein	
50	BHE75_04585	*oppA2*	2,4-dichlorophenol 6-monooxygenase	Unknown
51	BHE75_04586		Hypothetical protein	
52	BHE75_04587	*bphD*	2-hydroxy-6-oxo-6-(2′-aminophenyl)hexa-2,4-dienoic acid hydrolase	2-hydroxy-6-oxo-6-(2′-aminophenyl)hexa-2,4-dienoic acid hydrolase (*or meta*- ring fusion product hydrolase)
53	BHE75_04588	*BphR1*	GntR-type σ^54^-dependent transcriptional regulators	Transcriptional regulatory protein
54	BHE75_04589	*BphR2*	XylR_N-type σ^54^-dependent transcriptional regulator	Transcriptional regulatory protein
55	BHE75_04590	*bphA1*	Benzene 1,2-dioxygenase subunit alpha	Biphenyl 1,2-dioxygenase alpha subunit
56	BHE75_04591	*bphA2*	Biphenyl dioxygenase subunit beta	Biphenyl 1,2-dioxygenase beta subunit
57	BHE75_04592	*bphA3*	Biphenyl dioxygenase ferredoxin subunit	Biphenyl dioxygenase ferredoxin subunit
58	BHE75_04593	*bphA4*	Benzene 1,2-dioxygenase system ferredoxin–NAD(+) reductase subunit	Biphenyl dioxygenase ferredoxin-reductase subunit
59	BHE75_04594	*bphB*	Cis-2,3-dihydrobiphenyl-2,3-diol	Cis-2,3-dihydrobiphenyl-2,3-diol dehydrogenase
60	BHE75_04595	*bphH2*	2-keto-4-pentenoate hydratase	2-keto-4-pentenoate hydratase
61	BHE75_04596		Hypothetical protein	
62	BHE75_04597		Tn3 transposase DDE domain protein

The *ben/cat* operons contained genes for the transformation of BA to catechol (*benABCD*) and then finally to acetyl-CoA and succinyl-CoA via the *ortho* - cleavage pathway (*catABC* and *pcaDFIJ*). A LysR-type transcriptional regulatory protein (*benR*) was identified at the 5′ end of each operon and was inversely oriented to other genes (Table 1, Fig. 1). The two *ben/cat* operons showed similar gene organization with the sole differences being: (i) the *pcaDF* gene organization; in operon 1 *pcaF* preceded *pcaD* and (ii) the position of *benC*, which was located between *benB* and *benD* in operon 1, while a putative *benC* (Ferredoxin–NAD(P)(+) reductase) was localized at the 3′ end of operon 2 (Fig. 1). The role of each of the *ben/cat* operons was further elucidated by proteomic and transcription analysis and is discussed in the relevant sections.

Operon 3 contained genes encoding a flavin-dependent monoxygenase (*oppA1*), a 2,3 dihydroxy, 1,2-dioxygenase (*oppC*) and two *meta*-ring fusion product hydrolases (o*ppD1* and *oppD2*) (Table 1, Fig. 1). These genes composed a gene cluster (*oppD1ACD2*), potentially homologous to the *hbpCAD* gene cluster driving the upper part of the metabolic pathway of OPP in *P. azelaica* HPB1^26^. The translated products of *hbpCAD* genes showed 32–45% identities and 51–62% positives to their orthologs OppD1ACD2. Downstream of *oppD1ACD2*, genes encoding the complete lower biphenyl (*bph*) pathway were found, including a 2-keto-4-pentanoate hydratase (*bphH1*), an acetaldehyde dehydrogenase (*bphJ*) and a 4-hydroxy-2-oxovalerate aldolase (*bphI*). Denef *et al*.^27^ have shown that a similar gene cluster in *Burkholderia xenovorans* LB400 was responsible for the transformation of 2-hydroxypenta-2,4-dienoate (produced by the hydrolysis of the 2-hydroxy-6-oxo-6-phenyl–2,4-hexadienoic acid) to acetaldehyde and pyruvate. A transcriptional regulatory gene (*oppR*) showing high homology to a XylR_N superfamily of σ^54^-dependent transcriptional regulators was identified upstream of *oppD1* (Table 1). This is in accordance with the regulation of the *hpbCAD* operon in *P. azelaica* which was operated by a σ^54^-dependent XylR/DmpR transcriptional regulator^26^.

Operon 4 encodes an incomplete upper *bph* pathway (for the transformation of biphenyl to BA and 2-hydroxypenta-2,4-dienoate). This consists of *bphA1A2A3A4* (multi-component biphenyl dioxygenase), *bphB* (*cis*-biphenyl dihydrodiol dehydrogenase) and *bphD* (2-hydroxy-6-oxo-6-(2′-aminophenyl)hexa-2,4-dienoic acid hydrolase), only missing *bphC* (2,3-dihydroxybiphenyl dioxygenase). The latter is known to be responsible for the transformation of biphenyl-2,3-diol to 2-hydroxy-2,4-pentadienoate and benzoate^28^. The upper *bph* pathway is ubiquitous in soil bacteria^29^ and is usually found on transposable elements^30^. A second *bphH2 and* a gene encoding a second flavin-dependent monoxygenase (putative *oppA2*) were both located at the 5′ end of this operon. Two putative transcriptional regulatory genes were identified upstream of the *bph* genes (*bphR1R2*).

Phylogenetic analysis of the proteins encoded in the four catabolic operons provided insights into their origin and evolution. Operon 1 proteins (i.e. BenA, CatA, PcaD presented as indicative enzymes of the whole operon) clustered within the genus *Sphingomonas* and more specifically with proteins from a homologous operon of the HCH-degrading strain *Sphingomonas* MM1^31^ (Supplementary Fig. S1). Their orthologs from operon 2 clustered with proteins found in bacteria from the wider sphingomonad complex (*Sphingobium* and *Novosphingobium*) (Supplementary Fig. S1). In contrast, proteins encoded in operons 3 (OppC, OppD1D2) and 4 (BphA3, BphB) were associated with taxonomically distant bacteria (*Burkholderiaceae*, *Streptomyces* sp.) (Supplementary Fig. S2). These results suggest that the *ben/cat* operon 2 was laterally acquired by a member of the sphingomonad complex via horizontal gene transfer, in line with its flanking by tranposases. Whereas operons 3 and 4 constitute a patchwork assembly, with operon 4 probably still undergoing evolution as indicated by the lack of the *bphC* gene.

**Figure 2:**
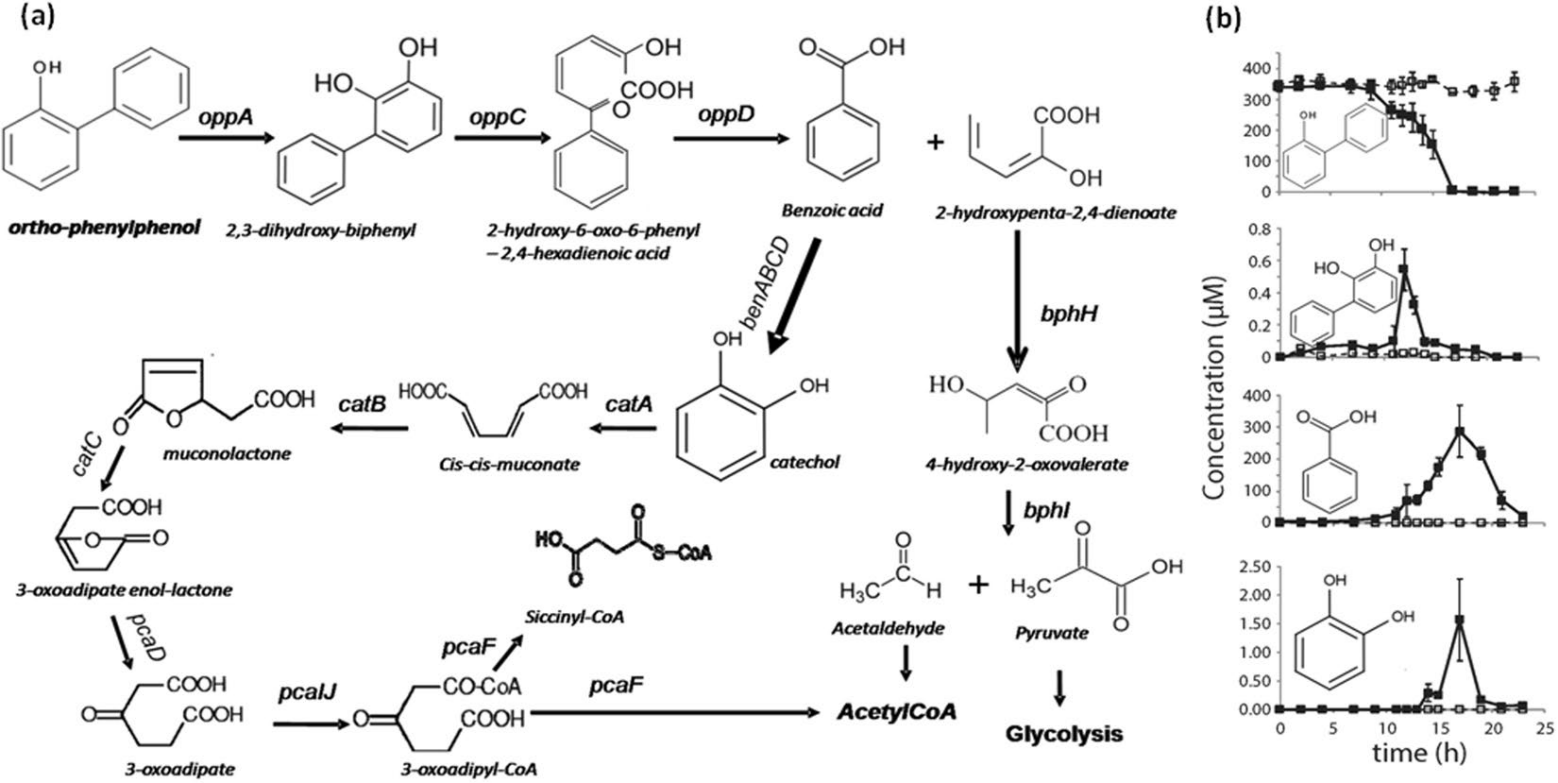
(**a**) The proposed metabolic pathway of *ortho*-phenylphenol (OPP) depicted by the genomic analysis of the *Sphingomonas haloaromaticamans* strain P3; (**b**) the degradation of OPP by strain P3, and the formation of 2,3-dihydroxybiphenyl, benzoic acid (BA) and catechol in inoculated (■) and non-inoculated samples (□). Each value is the mean of three replicates ± the standard deviation.

Based on the genomic analysis of the *S. haloaromaticamans* strain P3, a putative metabolic pathway is proposed (Fig. 2a), where OPP is transformed to BA and 2-hydroxypenta-2,4-dienoate (operon 3). These are further transformed to Krebs cycle intermediates through the *ben/cat ortho* cleavage pathway (operons 1 or 2) and the lower *bph* pathway (operon 3), respectively. The pathway proposed is similar to the metabolic pathway of OPP by the *P. azelaica* strain HBP1 with the sole difference of the *meta* cleavage of BA operated in strain HBP1^5^.

### Analytical determination of selected metabolic products of OPP

Key intermediate transformation products of the proposed metabolic pathway of OPP were detected by HPLC. The degradation of OPP by *S. haloaromaticamans* was rapid and concurred with the formation of BA, which peaked at 17 h and rapidly reduced thereafter (Fig. 2b). 2,3-dihydroxybiphenyl, the first metabolic product of the pathway, and catechol, the product of BA oxidation, were transiently detected at low levels. The transient formation of 2,3-dihydroxybiphenyl and catechol is probably a function of the capacity of *S. haloaromaticamans* to transform these intermediates at rates higher than that of their formation. Previous studies with *P. azelaica* HPB1 and other catechol-degrading bacteria^26,32^ have suggested that this metabolic strategy is a clever mechanism to overcome the toxicity of such metabolic intermediates to bacterial cells.

### Proteomic analysis

**Figure 3:**
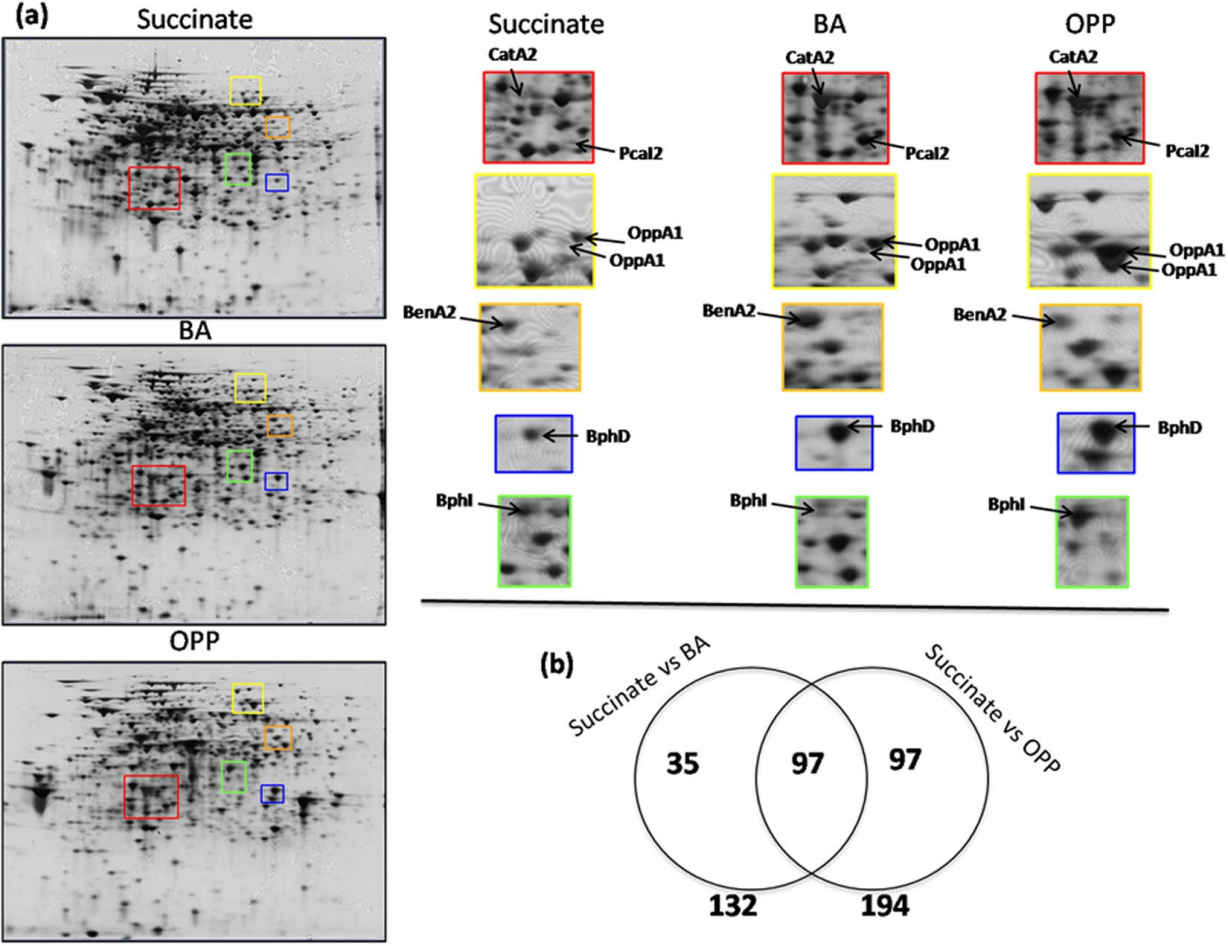
(**a**) 2D gels of the proteome of *S. haloaromaticamans* cells grown in MSMN + CA + ortho-phenylphenol﻿ ﻿(OPP), + benzoic acid (BA) or + Succinate. Colored frames on the 2D gels indicate regions where relevant catabolic proteins are located and the enlarged frames offer a focus on the intensity of spots associated to selected catabolic proteins among the different treatments; (**b**) Venn diagram representing the number of protein spots which showed differential expression in BA- vs succinate - growing cells (35) or in OPP - compared to succinate - growing cells (97). Furthermore, 97 common proteins showed differential expression in OPP- and BA-growing cells vs succinate-growing cells.

Proteomic analysis explored the role of the catabolic operons on the microbial transformation of OPP and provided an overview of the cell adaptation responses of the P3 strain during the degradation of OPP and/or BA. The bacterium was grown under selective conditions with OPP, BA and succinate as sole carbon sources and its proteome was analyzed at the mid-log phase of growth. This coincided with the near complete dissipation of OPP and BA (13 h) (Supplementary Fig. S3). 2D gel-based proteomic analysis identified 229 protein spots that were differentially expressed in the presence of OPP and/or BA compared to succinate (Fig. 3). Among these, 97 and 35 proteins showed differential expression only in the presence of OPP or BA respectively, compared to succinate. In addition 97 proteins showed differential expression in the presence of OPP and BA vs succinate (Fig. 3). Differentially expressed protein spots were excised, sequenced, and annotated. Quantitative and sequencing data of the proteins identified in the proteome of strain P3 are given in Supplementary Tables S1 and S2, respectively, and the position of each protein spot in the 2D-gels is given in Supplementary Fig. S4.

#### OPP catabolic proteins

Thirteen spots were associated with proteins having a putative role in OPP transformation. They were all up-regulated in the presence of OPP and/or BA and showed homology to translated genes from operons 2, 3 and 4 (Table 2). Proteins which were highly up-regulated only in the presence of OPP included (i) a flavin-dependent monoxygenase OppA1, (ii) the *meta*-ring fusion product hydrolases OppD1 and BphD and (iii) a 4-hydroxy-2-oxovalerate aldolase BphI (Fig. 3). These enzymes are involved either in the upper part of the OPP metabolic pathway (OppA1, OppD1, BphD) or in the transformation of 2-hydroxypenta-2,4-dienoate (BphI), both being modules of the OPP pathway that do not involve BA (Fig. 2a). BenA and BenB were up-regulated only in the presence of BA (Fig. 3), while CatA, PcaI and PcaF were up-regulated in the presence of both OPP and BA (Fig. 3), in line with their role in the downstream metabolism of BA in the OPP (BA is an intermediate metabolite) and BA treatments.

**Table 2:** Differentially expressed proteins with a putative role in the catabolism of *ortho*-phenylphenol (OPP) and benzoic acid (BA) in the proteome of *S. haloaromaticamans* grown on OPP or BA compared to cells grown on Succinate (Succ).

Spot No.	Protein identification^a^	Protein name	BA/Succ	OPP/Succ	Gene locus code^b^
7	Flavin-dependent monoxygenase	OppA1	4.55***	51.35*	BHE75_4573
15	Flavin-dependent monoxygenase	OppA1	5.06*	17.89*	BHE75_4573
2	2-hydroxy-6-oxononadienedioate hydrolase	OppD1	2.88	284.07**	BHE75_4572
79	2-hydroxy-6-oxo-6-(2′-aminophenyl)hexa-2,4-dienoic acid hydrolase	BphD	8.9**	704.02**	BHE75_4587
11	Benzoate dioxygenase large subunit	BenA2	3.51**	1.2	BHE75_4555
121	Benzoate dioxygenase small subunit	BenB2	13.38**	3.16	BHE75_4554
32	Catechol 1,2-dioxygenase	CatA2	3.99**	2.49*	BHE75_4556
34	Catechol 1,2-dioxygenase	CatA2	2.63*	1.81	BHE75_4556
76	Catechol 1,2-dioxygenase	CatA2	6.34***	8.85***	BHE75_4556
145	Catechol 1,2-dioxygenase	CatA2	1.9**	2.79**	BHE75_4556
112	putative succinyl-CoA:3-ketoacid CoA transferase subunit A	PcaI2	27.87***	32.82***	BHE75_4552
3	4-hydroxy-2-oxovalerate aldolase	BphI	0.85	2.94*	BHE75_4580
18	4-hydroxy-2-oxovalerate aldolase	BphI	0.25	4.11**	BHE75_4580

The spot intensity ratios of the BA/Succ and OPP/Succ are shown as a measure of proteins differential expression.

^a^Protein annotation based on homology with the translated genome of *S. haloaromaticamans* P3.

^b^The locus number of the gene which showed the highest homology with the sequenced protein spot.

#### Stress-related proteins

Several proteins involved in bacterial stress response were up-regulated in OPP- and BA-grown cells (Supplementary Table S3). The significant up-regulation of alkyl hydroperoxide reductase and superoxide dismutase, major scavengers of hydroxyperoxides, in the OPP-growing cells suggests the activation of a mechanism to cope with the oxidative stress induced by OPP or its chemically reactive intermediates (i.e. 2,3-dihydroxybiphenyl and catechol) which are known to produce reactive oxygen species and damage cell membranes^33^. Alkyl hydroperoxide reductase was among the more strongly up-regulated proteins in *P. putida* KT2440 cells growing in BA, p-hydroxybenzoate, vaniline and phenylethylamine compared to its expression in succinate growing cells^34^. The concurrent up-regulation of chaperons and chaperonins in the OPP and BA-grown cells is in line with the parallel activation of a stress-response mechanism. Chaperons (DnaK) and chaperonins are essential for the survival of bacteria under stress conditions since they facilitate the correct folding of denaturated proteins in the cytosol^35,36^. The mobilization of stress response mechanisms by *S. haloaromaticamans* cells during degradation of OPP and BA suggests that these compounds are not preferred growth substrates. Previous proteomic analyses with other xenobiotic-degrading bacterial strains have also noted a stimulation of the stress response mechanisms during degradation of BA^36^, phenanthrene^37^, and phenylurea herbicides^38^.

#### Transporters and membrane proteins

Up-regulation of transporters (i.e. ABC transporter) and proteins involved in membrane permeability and stability (i.e. TonB, pesticin receptor, YceI) was detected in the proteome of OPP-grown cells (Supplementary Table S3). This is in line with previous studies with other xenobiotics-degrading strains like *Pseudomonas putida* KT2440^39,40^. Dominquez-Cuevas *et al*.^33^ showed that the primary effect of toluene to *P. putida* KT2440 is at the cell envelope level, which then leads to reciprocal oxidative damage and mobilization of the stress response system of the bacterial cell. Flagella-domain related proteins were also highly up-regulated in OPP- and BA-grown cells (Supplementary Table S3). Nikodinovic-Runic *et al*.^41^ also observed an up-regulation of the biosynthesis of flagella-associated proteins upon exposure of a *P. putida* to styrene in N starvation conditions. This was attributed to a general stimulation of bacterial motility in response to environmental perturbations like exposure to organic pollutants.

#### Proteins involved in energy production

A large number of proteins involved in energy production (i.e. ATP synthases, ubiquinol-cytochrome c reductase, electron transfer flavoprotein, NADH dehydrogenase/NAD(P)H nitroreductase, 2,3-bisphosphoglycerate-dependent phosphoglycerate mutase) and in the synthesis of biomolecules (i.e. aspartate aminotransferase, 30S and 50S ribosomal proteins, elongation factors) were up-regulated in OPP- and/or BA-grown cells (Supplementary Table S3). Previous studies with *P. putida* KT2440 also noted a significant up-regulation of proteins with a similar biosynthetic role in cells grown with xenobiotics^34,42^. This is probably a response for the *de novo* synthesis of proteins to counterbalance the toxicity of OPP or its intermediates and the parallel increase in needs of the growing cells. Jang *et al*.^43^ investigated the cellular response of *Staphylococcus aureus* to OPP and observed a strong over-expression of genes encoding ribosomal proteins probably related to an overall stimulation of the translation process triggered by stress conditions.

Overall, proteomic analysis further supported the metabolic pathway deduced by the genomic analysis and showed that *S. haloaromaticamans*, despite its high degradation efficiency, mobilizes its stress-related cellular mechanisms as a response to the potential toxicity of OPP or its transformation intermediates to bacterial cells.

### Transcription analysis of catabolic genes

The expression pattern of all putative catabolic genes identified in the genome and especially those in the proteome of *S. haloaromaticamans* was determined via RT-q-PCR. This allowed further verification of the role of each of these genes in the metabolism of OPP. Thus, the bacterium was grown in OPP or BA and the expression of these selected genes was determined, along with the degradation of these compounds, and compared to their expression in succinate-grown cells.

#### Upper OPP pathway

**Figure 4:**
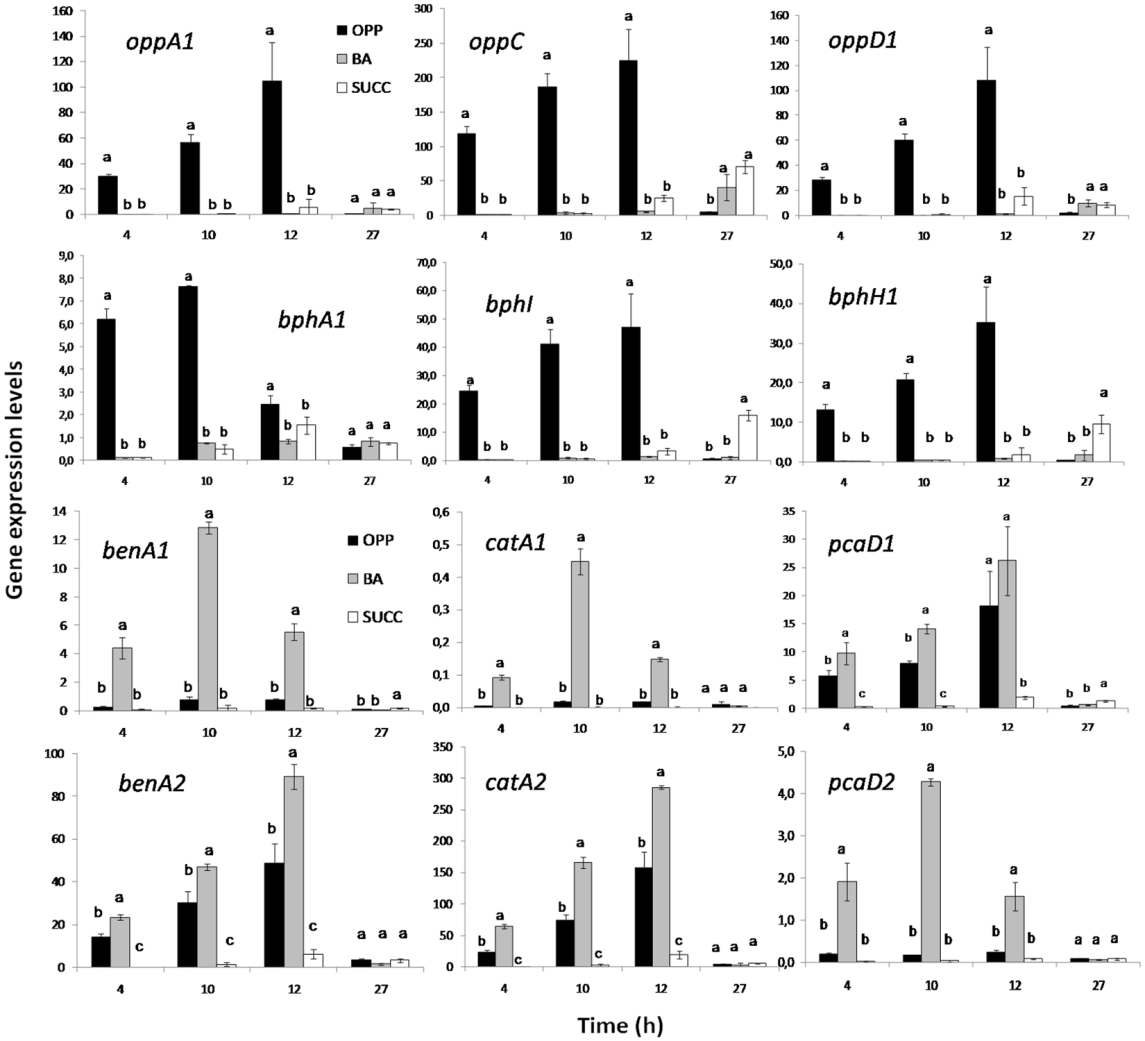
The transcription patterns of selected genes involved in the upper metabolic pathway of *ortho*-phenylphenol (OPP) (*oppA1, oppC, oppD1*), in the upper and lower *bph* pathway (*bphA1,bphI, bphH1*) and in the *ortho* cleavage of benzoic acid (BA) (*ben/cat* pathway) by *Sphingomonas haloaromaticamans* strain P3 growing on MSM + CA + OPP, + BA or + Succinate (Succ). Within each time point bars designated by the same letter are not significantly different at the 5% level. The transcription patterns of the remaining catabolic genes are given in Supplementary Figs S5–S8.

The expression of all genes with a putative catabolic role in the upper part of the OPP pathway (*oppD1A1CD2, oppA2*) showed similar patterns with significantly higher expression (*p* < *0.05*) in the presence of OPP compared to BA and succinate (Fig. 4 and Supplementary Fig. S5). Their expression significantly increased up to 12-h, which coincided with the near complete degradation of OPP (Supplementary Fig. S3) and dropped to levels similar to the other two treatments by the end of the study (27 h) (Fig. 4). The only exception was *oppA2*, whose expression did not differ between the different treatments (Supplementary Fig. S5). These findings suggest that from the two flavin-dependent monoxygenases found in the genome of *S. haloaromaticamans* it is OppA1 that drives the initial hydroxylation of OPP. OppA2 might be involved in the hydroxylation of aromatic compounds other than OPP and BA, however its function to date remains unknown. Among genes of the upper OPP pathway *oppC* showed the highest expression levels (Fig. 4). This might point to a metabolic strategy aiming to prevent the accumulation of the toxic intermediate 2,3-dihydroxybiphenyl as suggested above. A similar transcription regulation strategy was observed in the other OPP-degrading strain *P. azelaica* HBP1^6^.

#### Upper and lower biphenyl pathway

Genes *bphA1A2A3A4*, *bphB* and *bphD* (operon 4), showed a significantly higher expression in the presence of OPP compared to BA- and succinate-grown cells (Fig. 4, Supplementary Fig. S6). The expression levels of these genes were much lower compared to the respective genes of the upper OPP pathway (*oppD1A1CD2*), suggesting an auxiliary role in the degradation of OPP. This is in agreement with the low substrate specificity of biphenyl dioxygenases^44,45^ and the structural resemblance of OPP and biphenyl, whose transformation converges on the same intermediates acting as weak effectors of the *bph* pathway. In contrast to the other genes of the operon, *bphD* showed expression levels equivalent to *oppD1* from operon 3 (Supplementary Fig. S6), in line with their significant up-regulation in the proteome of OPP-growing cells (Table 2). The high levels of co-expression of *oppD1D2* and *bphD* might be a strategy for accelerating the transformation of the *meta*-cleavage product or equal affinity for the common substrate produced by the transformation of OPP and biphenyl.

Genes *bphH1* and *bphI* showed significantly higher expression levels during the first 12-h of OPP degradation compared to their expression in BA- and succinate-grown cells (Fig. 4). The *bphH2* gene (operon 4) showed significantly higher expression when grown on OPP (Supplementary Fig. S6), but still much lower compared to its ortholog (*bphH1*) in operon 3, further verifying the prime role of operon 3 in the transformation of OPP and its intermediates like 2-hydroxypenta-2,4-dienoate.

**Figure 5:**
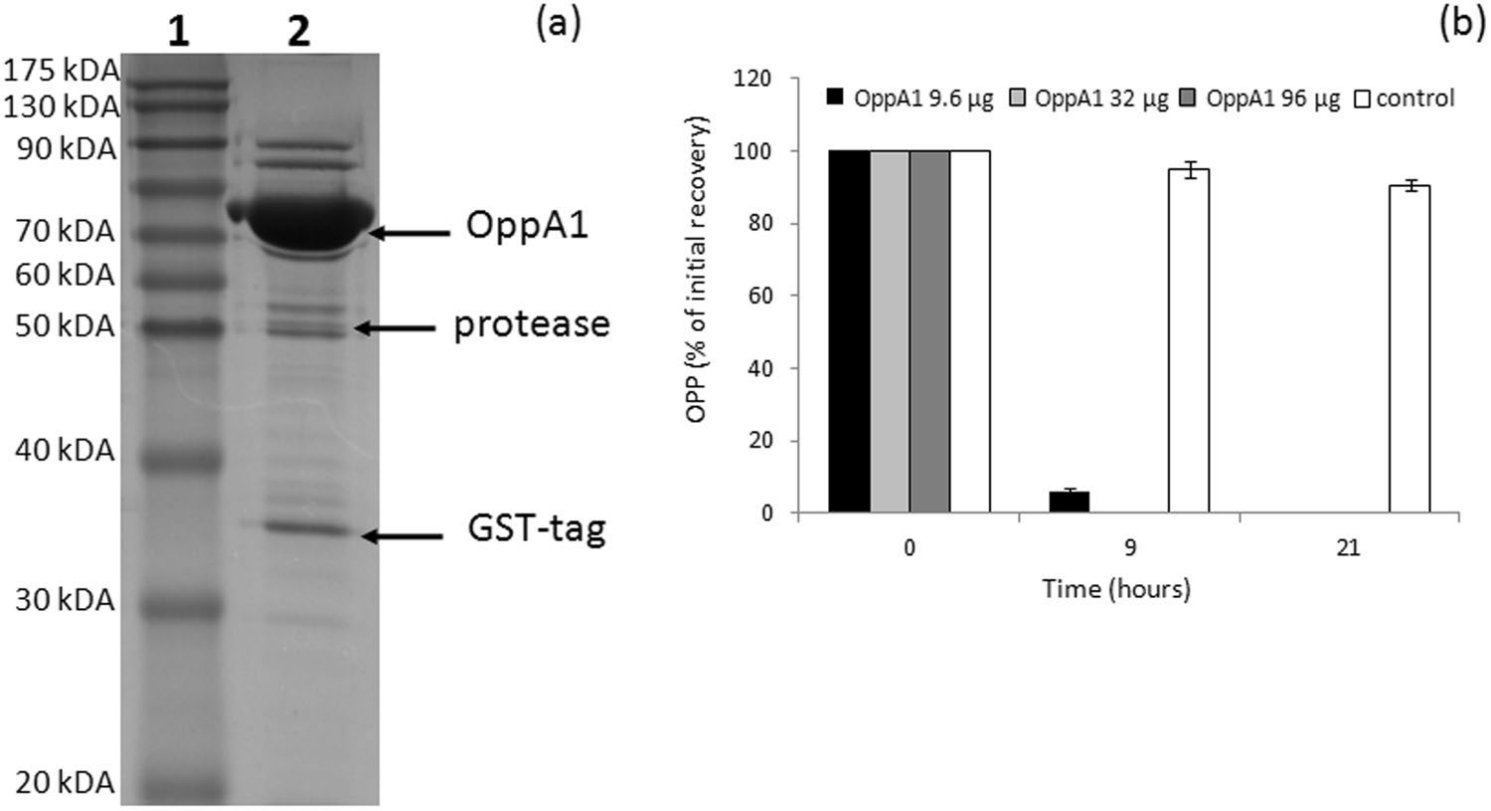
SDS-PAGE analysis of the flavin-dependent monoxygenase of *ortho*-phenylphenol (OppA1) isolated by *Sphingomonas haloaromaticamans* strain P3. Lane 1: Molecular mass ladder; Lane 2: Elution from a Protino Glutathione Agarose 4B column, and after in column digestion with protease, where the bands of OppA1, protease and GST-tag are indicated with arrows; (**b**) OPP degradation by different starting amounts of the purified OppA1. Each value is the mean of three replicates ± the standard deviation.

#### Ben/cat pathway (lower OPP pathway)

All genes from the same *ben/cat* operon showed uniform expression patterns. Genes from both operons showed significantly higher expression in BA- (*p* < *0.05*) compared to OPP- and succinate-grown cells (Fig. 4; Supplementary Fig. S7). Whereas OPP induced a significant increase (*p* < *0.05*) in the expression levels only of the operon 2 genes. When the expression level of orthologous genes of the two *ben/cat* operons were compared, a significantly higher expression of all genes from operon 2 was evident, suggesting that *S. haloaromaticamans* activates operon 2 for the metabolism of BA formed by OPP transformation. Considering the localization of operon 1 in the bacterial chromosome, it is tempting to assume that this operon is utilized by the bacterium for the consumption of biogenic aromatic organic acids, like substituted benzoic and cinnamic acids with methoxy or hydroxy substituents^46^, which thrive in the soil environment. B*en/cat* operons are ubiquitous in soil bacteria playing a central role in the degradation of biogenic aromatic compounds encountered in soil^47^. Alternatively, *ben/cat* operon 1 might be regulated differently depending on the concentration of BA or the growth stage of the bacterium. Denef *et al*.^48^ showed that the biphenyl-degrading strain *Burkholderia xenovorans* LB400 could concurrently utilize two or three pathways to transform BA depending on the initial substrate (BA or biphenyl) and its growth stage.

The presence of two peripheral (upper *opp* and upper *bph*) and two central catabolic pathways (*ben/cat* and lower *bph*) in *S. haloaromaticamans* is contrary to the modular construction of catabolic pathways found in most xenobiotic-degrading bacteria, where several peripheral metabolic pathways are funnelling their products into a single central catabolic pathway^49^. The phylogenetic classification of the genes of the two *ben/cat* operons and the flanking of operon 2 by transposable elements supports its lateral acquisition from a phylogenetically close bacterium through horizontal gene transfer, rather than through duplication and gradual evolution of operon 1, a strategy commonly utilized by other oligotrophs (i.e. Arthrobacters)^50^. Sphingomonads are known to utilize horizontal gene transfer as a major mechanism to expand or optimize their catabolic capacities^17,18,21^. We suggest that *S. haloaromaticamans* has evolved its capacity to metabolize OPP by gradual acquisition and assemblage of operons 2 and 3 from taxonomically close and distant bacteria, respectively. While the acquisition of operon 3 by *S. haloaromaticamans* was a key step towards the development of its capacity to transform OPP, the acquisition of operon 2 is most probably an optimization step towards a more efficient metabolism of BA.

#### Transcriptional regulatory proteins

*BenR1* was significantly up-regulated only in the presence of BA, whereas *benR2* was significantly up-regulated when grown in OPP and BA (Supplementary Fig. S8), in line with the expression patterns of catabolic genes in their respective operons. Up-regulation of both *benR1* and *benR2* was observed only at 27 h (after completion of BA degradation) indicating a transcriptional repressor activity. Vasely *et al*.^51^ reported a repressor activity of *catR* on the *catABC* locus of a *Rhodococcus erythropolis* strain, whereas other studies have shown that *catR* acts as an activator of the *ortho*-cleavage pathway^52^. The presence of a single regulatory protein BenR for both segments of the pathway (*ben* and *cat*) has been reported previously in xenobiotics-degrading sphingomonads^31^ and it was identified as an effective transcription regulation mechanism induced by both BA and *cis*, *cis*-muconate^53^.

The putative transcriptional regulatory genes of operons 3 and 4 showed higher expression levels (*p* *<* *0.05*) in the presence of OPP (Supplementary Fig. S8), although they exhibited diverse expression patterns. In operon 3, *OppR1*, a XylR_N-type σ^54^-dependent transcriptional regulator, showed a significant increase in its expression after completion of OPP degradation (27 h) (Supplementary Fig. S8), suggesting a repressor activity. In contrast, the *hbpR* of *P. azelaica* HBP1 was a transcriptional activator of *hbpCAD*^26^. In operon 4, *bphR1* and *bphR2*, GntR-type and XylR_N-type σ^54^-dependent transcriptional regulators respectively, showed increasing expression levels (*p* *<* *0.05*) during degradation of OPP suggesting a transcriptional activator regulatory role (Supplementary Fig. S8). Two-component regulatory systems are a common feature of biphenyl-degrading bacteria composed usually by a GntR family transcriptional regulator, acting either as an activator^27,54^ or as a repressor^28,55^, and a second LysR-type transcriptional regulator.

Overall, transcription analysis further verified the metabolic pathway of OPP, as initially depicted by the genomic and proteomic analysis, clarified the role of orthologous enzymes in the metabolic pathway of OPP and provided novel insights into the genetic networking regulating the metabolic pathway of the fungicide by *S. haloaromaticamans* strain P3.

### Heterologous expression of the flavin-dependent monoxygenase OppA1

Proteogenomic and transcription analysis pointed to *oppA1* (BHE75_04573) as responsible for the initial hydroxylation of OPP. The flavin-dependent monoxygenase encoded by this gene was isolated via heterologous expression in *E. coli* and purified (Fig. 5a). The isolated protein had an estimated molecular mass of *ca*. 68 kDa as determined by SDS-PAGE, in line with its predicted molecular mass based on its amino acid sequence. The recombinant enzyme was functional and degraded OPP *in vitro* in less than 9 h when 32 or 96 μg were added in the reaction, and in less than 21 h when a lower enzyme amount was used in the *in vitro* test (9.6 μg; Fig. 5b). These results confirmed that the monoxygenase encoded by *oppA1* is responsible for the initial hydroxylation of OPP in the metabolic pathway of OPP by *S. haloaromaticamans*. Further analysis will focus on the detailed characterization of OppA1, considering the high industrial importance of such monoxygenases in the hydroxylation of aromatic compounds whose hydroxylation by chemical means requires harsh conditions and toxic reagents.

**Figure 6:**
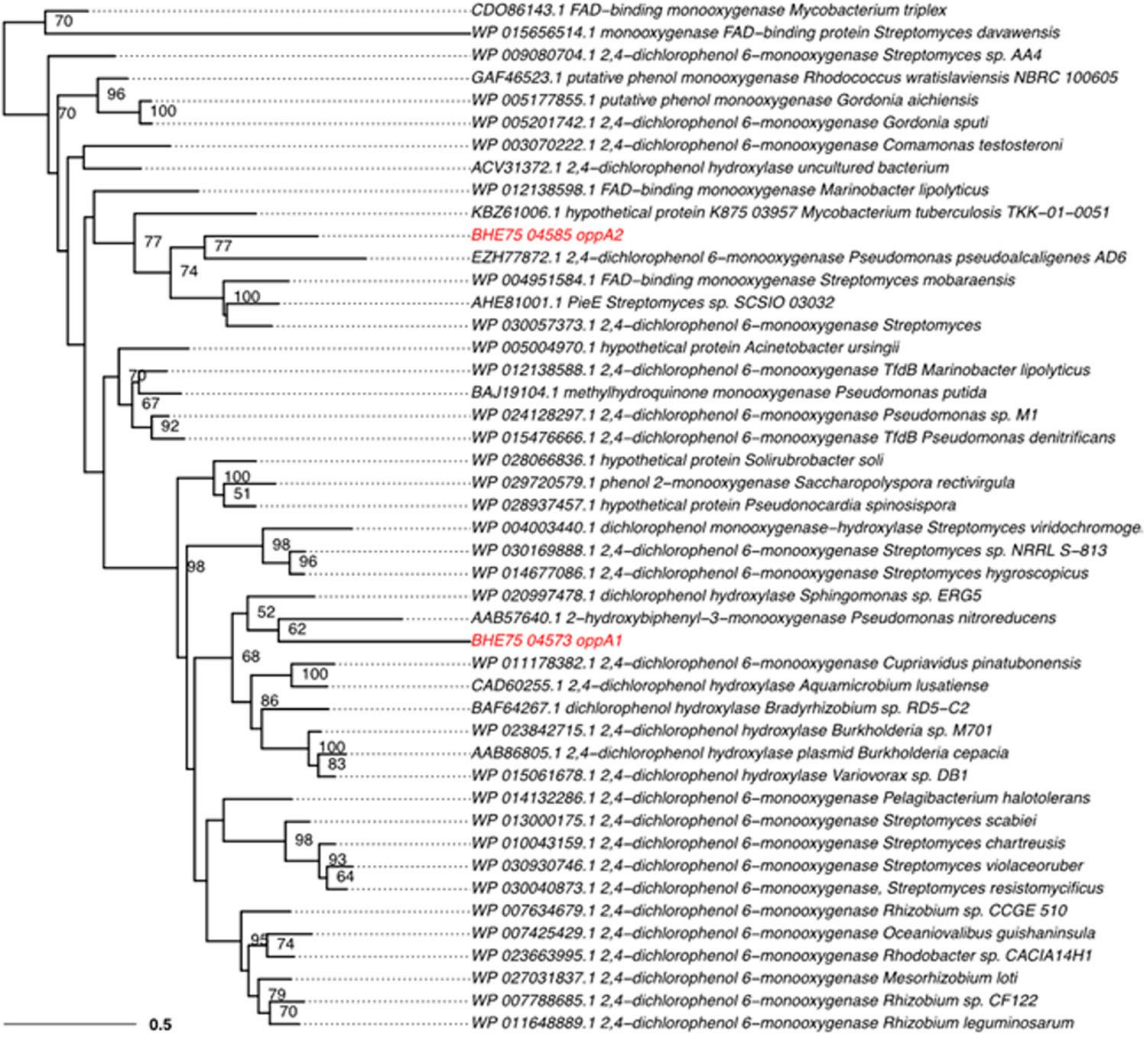
Maximum likelihood tree of the two flavin-dependent monoxygenases OppA1 and OppA2 found in the genome of the *S. haloaromaticamans* using 1000 bootstrap replicates and the Le-Gascuel (LG^67^) model with gamma rate heterogeneity and accounting for invariable sites.

Phylogenetic analysis of OppA1 revealed that it is closely affiliated to other 2,4-dichlorophenol-6-monoxygenases and formed a well-supported clade with the 2-hydroxybiphenyl-3-monoxygenase, responsible for the initial hydroxylation of OPP by *P. azelaica* HBP1 (formely known as *Pseudomonas nitroreducens*), and a dichlorophenol hydroxylase from another *Sphingomonas* isolate (Fig. 6). All 2,4-dichlorophenol-6-monoxygenases belong to the class A of flavoprotein monoxygenases (EC.1.14.23.13), are known to hydroxylate various aromatic compounds and participate in important biosynthetic and transformation pathways^56^. The 2-hydroxybiphenyl-3-monoxygenase of *P. azelaica* HBP1 is one of the most well studied enzymes of the class A flavin-dependent monoxygenases^7,8^ and it has been used for the industrial scale production of 3-substituted catechols^57^.

## Concluding Remarks

The metabolic pathway of OPP by the *S. haloaromaticamans* strain P3 was elucidated using a combination of genomics and proteomics, whose results were further verified by transcription and chromatographic analyses. OPP is transformed, through the upper metabolic pathway, to BA and 2-hydroxypenta-2,4-dienoate. The former is further metabolized via the *ortho* cleavage pathway and the latter is transformed via the lower *bph* pathway, both to Krebs cycle intermediates. The key enzyme of the pathway, a flavin-dependent monoxygenase, was isolated and its activity against OPP was verified *in vitro*. Solid evidence suggest that the catabolic operons controlling the transformation of OPP are part of a 55-kb transposon which was probably acquired by *S. haloaromaticamans* via horizontal gene transfer. Proteomic analysis revealed the activation of a stress-related response by *S. haloaromaticamans* during degradation of OPP which was echoed in the up-regulation of associated functions like protein synthesis, energy production, motility ﻿and membrane transportation.

## Materials and Methods

### Bacterial strain, growth conditions and chemicals

The *S. haloaromaticamans* strain P3, using OPP as a carbon source, was routinely cultivated in a mineral salts medium supplemented with nitrogen and casamino acids (0.15 g L^−1^) (MSMN + CA)^3^. The medium was amended with filter sterilized aqueous solutions of OPP (200 mg L^−1^), BA (200 mg L^−1^) and succinate (2000 mg L^−1^). Bacterial growth was determined by optical density at 600 nm (OD_600_). Analytical standards of OPP (99.9%), BA (99.5%), 2,3-dihydroxybiphenyl (≥98%), catechol (≥99%) were purchased from Sigma-Aldrich (St Louis, USA) and succinate (99%) from PanReac-AppliChem (St. Louis, USA).

### Genomic analysis of *S. haloaromaticamans*

Total DNA was extracted from a fresh culture of *S. haloaromaticamans* with the Purelink Genomic DNA Mini kit (Invitrogen Life Technologies, USA) and quantified by Qubit (Fisher Scientific, USA). Sequencing was performed by Illumina MiSeq with a 2 × 300 bp paired-end (insert ~550 bp) and a 2 × 300 bp mate-pair (insert ~3000 bp) runs. Genome assembly was performed with Allpaths-LG v50960^58^ using the default parameters. Genome completeness and purity was checked with the CheckM v0.9.6 software suite^59^ and annotation of the resulting contigs was performed with Prokka v1.10^60^ as described in details in the Supplementary Information (see Section SI 1.1.).

### Phylogenetic analyses of catabolic enzymes

The translated sequences of the catabolic genes identified via genomic analysis were subjected to maximum likelihood phylogenies using the RAxML software v8.1.24^61^ as described in details in the Supplementary Information (see section SI 1.2.).

### Proteomic analysis of *S. haloaromaticamans*

#### Experimental set up and crude protein extraction

Triplicate cultures of MSMN + CA + OPP (50 mg L^−1^), + BA (62 mg L^−1^) or + succinate (100 mg L^−1^) were inoculated with *S. haloaromaticamans* as described above, aiming to equivalent carbon concentration in all treatments (42.5 mg C L^−1^). Duplicate non inoculated MSMN + CA + OPP or MSMN + CA + BA were also prepared. All samples were incubated in an orbital shaker at180 rpm and 26 °C. The degradation of OPP and BA and the growth of *S. haloaromaticamans* was determined at regular intervals by HPLC and OD_600_ measurements, respectively. When bacterial growth in all treatments reached the mid-log phase (OD_600_ = 0.15–0.16) bacterial cells were harvested by centrifugation (5000 rpm, 10 min, 4 °C) and used for protein extraction. Bacterial pellet was kept on ice and re-suspended in cold buffer A (50 mM tris-base, 100 mM NaCl, 10% glycerol, pH 7.5). Upon addition of 0.1% TRITON and 0.5 mM PMSF the cells were ultrasonicated on ice three times for 15 sec. Cell debris were removed by centrifugation (12000 rpm, 45 min, 4 °C).

#### 2-D proteomic analysis and protein identification by mass spectrometry

For 2D-PAGE separation crude protein extracts were further clarified, concentrated and protein pellet was solubilized at the desired volume of rehydration buffer as described before^62^. Protein concentration was determined according to Bradford^63^ using a Bio-Rad assay kit with BSA as standard. Protein extracts were analyzed by 2D-PAGE as described by Ainalidou *et al*.^64^. For each sample 30 μg of total soluble proteins were analysed. Proteins were first separated by isoelectric focusing using gel strips forming an immobilized non-linear pH gradient from 3 to 10 (pH 3–10 NL IPG strips, 11 cm; Bio-Rad) and then by SDS–PAGE using 12.5% Tris-HCl polyacrylamide gels (Bio-Rad) following standard procedures. For each treatment three biological replicates were run in parallel and silver stained. 2-DΕ gels were scanned with Bio-Rad GS-800 Calibrated Densitometer equipped with PDQuest Advanced 2-DΕ Gel Analysis software (version 8.1, Bio-Rad) as previously described^64^. Data were analyzed by one-way ANOVA (P ≤ 0.05) and means were compared using Student’s t-test (significance level 95%). The statistical significant differences were further combined by the quantitative 2-fold change of spot volume. Spots showing values in the ratios OPP/Succinate and BA/Succinate (volume intensity) lower than 0.5 or higher than 2 were excised from the 2D-PAGE gels and digested with trypsin (more details are available in the Supplementary Information section SI 1.3.).

Tryptic peptide mixtures were analyzed in a MALDI-TOF mass spectrometer (Autoflex-Speed, Bruker Daltonics). The protein identification was carried out by peptide mass fingerprinting on a locally installed Mascot-Server v 2.0 against the genome of *S. haloaromaticamans* P3 & Uniprot-Trembl databases. The mass error tolerance on the Mascot server was set to 25 ppm, methionine oxidation was considered as a variable modification and cystein carbamido methylation was considered as a fixed modification. Proteins not identified by MALDI-TOF analysis were reanalyzed by HPLC-tandem MS/MS (Thermo Scientific) as described in the Supplementary Information (see Section S.I. 1.3.).

### Transcription analysis

Bacterial pellet collected at 4, 10, 12 and 27 h during the proteomics experiment was stored at −80 °C and used for transcription analysis of putative catabolic genes. RNA was extracted with the Nucleospin RNA II kit (Macherey-Nagel, Germany). In most cases a DNAse treatment step (DNAse I, Amplification Grade, Invitrogen Life Technologies) was essential to remove DNA residues from extracted RNA. DNA-free RNA was then reverse transcribed to obtain cDNA (Superscript II, Invitrogen Life Technologies) using random hexamers (Takara, Japan).

Primers for the amplification of all putative catabolic genes of *S. haloaromaticamans* were manually designed with the program PrimerSelect™ (Lasergene®, DNASTAR) based on the genomic analysis of the studied strain (Supplementary Table S4). The primers were further checked for the potential formation of secondary structures and their specificity was validated *in silico* (Primer-BLAST, http://www.ncbi.nlm.nih.gov/tools/primer-blast/) and by PCR, using total DNA of strain P3, and sequencing of the PCR product obtained. Primers for the amplification of the *gyrB* gene of *S. haloaromaticamans* (reference gene in the transcription analysis) were also designed based on the genome of the strain P3 (Supplementary Table S4). RT-q-PCR thermocycling conditions and reagents are given in Supplementary Information (see section S.I. 1.4.). Quantification of gene expression was performed according to Pfaif^65^. Transcription analysis data were subjected to two-way ANOVA and significant differences were detected with the post-hoc Tukey test (p < 0.05). Statistical analysis was performed with the SPSS Statistics (IBM Corp. Version 21.0.) software.

### Isolation and in vitro assessment of the activity of flavin-dependent monoxygenase OppA1

Primers were designed to amplify the full length sequence of *oppA1* (oppAf 5′ACTCATGGATCCATGACTTCAGCAGTTCAAAAACCG3′ and oppAr 5′ACTCATCTCGAGTTAAGAAGCCGGCGTGAATTTTG3′). Underlined nucleotides indicate restriction sites for enzymes *Bam*HI and *Xho*I to facilitate ligation into the plasmid vector pGEX-6P-1 (N-terminal GST tag). Amplification of the putative *oppA* was performed in 50 μl reactions containing 1X Polymerase Buffer, 1.5 mM MgCl_2_, 0.2 mM of each dNTP, 0.5 μΜ of each primer, 1 U of Thermo Scientific Phusion High-Fidelity DNA Polymerase and 1 μl of total bacterial DNA. Amplification conditions were 98 °C for 30 sec, 30 cycles of 98 °C for 10 sec, 67 °C for 30 sec and 72 °C for 1 min, and final extension at 72 °C for 10 min. The product was purified, digested with *Bam*HI and *Xho*I and ligated into the pGEX vector digested with the same enzymes. Plasmids were transformed into *Escherichia coli* BL21(DH_3_) cells.

Eight hundred ml of *E. coli* transformed cell cultures were grown in LB + ampicillin (100 μg mL^−1^) in an orbital shaker (200 rpm) at 37 °C to an OD_600_ of 0.6. The recombinant enzyme was induced with IPTG (0.025 mM) and cells were harvested, 16 h after induction, by centrifugation at 8000 rpm at 4 °C for 7 min. Cells were re-suspended in Buffer A and lysed with ultrasonication (6 × 10 sec). The samples were centrifuged for 15 min at 14500 rcf at 4 °C. The soluble proteins in the supernatant were collected and purified by passage through pre-equilibrated Protino Glutathione Agarose 4B (Macherey-Nagel, Duren, Germany). The suspension was mixed gently at 4 °C for 2 h. The gel was centrifuged for 5 min at 500 *x*g and the supernatant was discarded. The gel was resuspended in 100 μl of buffer A, transferred to an appropriate chromatography column (Micro Bio-Spin™ Chromatography Columns) and washed successively with the solutions buffer A, buffer A + Triton (0.2%), buffer A and buffer A + DTT (10 mM). Then, the column outlet was closed with cap and the gel was incubated overnight at 4 °C with buffer A + 3 C protease (55 μg). After the incubation the eluate was collected and the purified protein was run with SDS-PAGE^66^. The gel was stained with Coomassie Brilliant Blue R-250 and destained with a solution of 30% methanol and 10% acetic acid in deionized water. The activity of the purified enzyme was tested in 1-ml reactions composed of 0.25 mM of OPP, 1 mM of NADH, 20 mM phosphate buffer (pH 7.5) and three protein amounts, 9.6, 32 and 96 μg. Triplicate reactions per protein level were prepared, while triplicate controls without protein were also included. The degradation of OPP was determined by HPLC-UV at 0, 9 and 21 h.

### Analytical detection of key OPP transformation products

Triplicate MSMN + CA + OPP (50 mg L^−1^) cultures were inoculated with *S. haloaromaticamans*. Triplicate non-inoculated controls were also included as abiotic controls. Immediately after inoculation and at hourly intervals thereafter the degradation of OPP and the formation of the putative metabolites 2,3-dihydroxybiphenyl, BA and catechol were determined by HPLC-UV. All chemicals were extracted from 0.5-mL aliquots and analysed as described before^3^.

### Data availability

The assembled genome of the strain P3 has been deposited at DDBJ/ENA/GenBank under the accession number MIPT00000000. All data generated or analysed during this study are included in this published article and its Supplementary Information files.

## Electronic supplementary material

Supplementary Information

Supplementary Table S1

Supplementary Table S2
