# Author Correction: Metabolic pathway and cell adaptation mechanisms revealed through genomic, proteomic and transcription analysis of a *Sphingomonas haloaromaticamans* strain degrading *ortho*-phenylphenol

**DOI:** 10.1038/s41598-018-22845-1

**Published:** 2018-03-12

**Authors:** Chiara Perruchon, Sotirios Vasileiadis, Constantina Rousidou, Evangelia S. Papadopoulou, Georgia Tanou, Martina Samiotaki, Constantinos Garagounis, Athanasios Molassiotis, Kalliope K. Papadopoulou, Dimitrios G. Karpouzas

**Affiliations:** 1 Department of Biochemistry and Biotechnology, University of Thessaly, Laboratory of Plant and Environmental Biotechnology, Viopolis, 41500 Larissa Greece; 2 University of South Australia, Future Industries Institute, Mawson Lakes, Australia; 3 Aristotle University of Thessaloniki, School of Agriculture, Thessaloniki, Greece; 4 Biomedical Sciences Research Center “Alexander Fleming”, Vari, 16672 Greece

Correction to: *Scientific Reports* 10.1038/s41598-017-06727-6, published online 25 July 2017

The original version of this Article incorrectly listed all author names in reverse. The author list now reads:

Chiara Perruchon, Sotirios Vasileiadis, Constantina Rousidou, Evangelia S. Papadopoulou, Georgia Tanou, Martina Samiotaki, Constantinos Garagounis, Athanasios Molassiotis, Kalliope K. Papadopoulou, Dimitrios G. Karpouzas

