# Supplementary material for: Metabolic pathway and cell adaptation mechanisms revealed through genomic, proteomic and transcription analysis of a Sphingomonas haloaromaticamans strain degrading ortho-phenylphenol
Source: Sci Rep. 2017 Jul 25;7:6449. doi: 10.1038/s41598-017-06727-6 (PMC5527002; doi:10.1038/s41598-017-06727-6)
Supplement: Supplementary file 1 — Supplementary Information [file 41598_2017_6727_MOESM1_ESM.pdf]

Metabolic pathway, cell adaptation mechanisms and a novel monoxygenase revealed through proteogenomic-transcription analysis of a *Sphingomonas haloaromaticamans* strain degrading *ortho*-phenylphenol

Chiara Perruchon,[1] Sotirios Vasileiadis,[2] Constantina Rousidou,[1] Evangelia S. Papadopoulou,[1] Georgia Tanou,[3] Martina Samiotaki,[4] Constantinos Garagounis,[1] Athanassios Molassiotis,[3] Kalliope K. Papadopoulou,[1] Dimitrios G. Karpouzas[1*].

[1] University of Thessaly, Department of Biochemistry and Biotechnology, Laboratory of Plant and Environmental Biotechnology, Viopolis 41500, Larissa, Greece

[2] University of South Australia, Future Industries Institute, Mawson Lakes, Australia

[3] Aristotle University of Thessaloniki, School of Agriculture, Thessaloniki, Greece

[4] Biomedical Sciences Research Center "Alexander Fleming", Vari 16672, Greece.

Supplementary Information (SI)

Materials and Methods

1.1. Genomic analysis of Sphingomonas haloaromaticamans *strain P3*

Genome completeness and purity was checked with the CheckM v0.9.6 software suite [1] and annotation of the resulting contigs was performed with Prokka v1.10 [2] using: Prodigal v2.6 [3] for protein coding gene calling; Barrnap v0.5 (http://www.vicbioinformatics.com/ software.barrnap.shtml) for the rRNA coding gene prediction; Aragorn v1.2.36 [4] for tRNA annotation; MinCED v0.1.6 (https://github.com/ctSkennerton/ minced/tree/master), the evolution of the CRT tool,

for clustered regularly interspaced short palindromic repeats (CRISPR) mining [5]; the protein-protein basic local alignment search tool (BLAST) v2.2.27+ [6] with an e-value cut-off of 10^{-9} while screening the UniProt database [7] full length sequences with protein or transcript evidence, and the profile alignment screening approach with hidden Markov models, HMMER v3.1 [8] searching the Pfam [9] and TIGRFAM [10] databases for protein coding gene annotation.

1.2. Phylogenetic analyses of catabolic enzymes

Closely related sequences of the *S. haloaromaticamans* genome predicted proteins of interest were retrieved from NCBI with BLASTv2.2.27+ and were clustered with Cdhit v4.6 [11] to reduce sequence redundancy. The sequences were then aligned with Muscle v3.8.31 [12], and improperly aligned and uninformative alignment blocks were removed using Gblocks v0.91b [13]. The remaining concatenated alignment blocks were subjected to maximum likelihood phylogenies with the RAxML software v8.1.24 [14] and 1000 bootstrap replicates using the best model according to ProtTest v3.4 [15] and the associated Akaike information criterion values (AIC). Tree visualization was performed using the APE v3.5 [16] and Phangorn v2.0.4 [17] R v3.3.1 [18] software packages.

1.3. Proteomic analysis of S. haloaromaticamans

2-D proteomic analysis and protein identification by mass spectrometry - trypsin digestion: Gel pieces were placed into 96-well microtiter plates, destained with 75 µL of 30mM potassium ferricyanide and 75 µl of 100 mM sodium thiosulfate solution and incubated for 3x10 min. Spots were rinsed twice with 150 µl of water and dried in a speed vacuum concentrator (MaxiDry Plus, Denmark). In-gel digestion was

performed with 0.01 μg μL-1 trypsin (Roche Diagnostics, Switzerland) for 16 h at room temperature. Next, 5 μL of 50% acetonitrile (ACN) containing 0.1% trifluoroacetic acid (TFA), were added to each dried gel piece and digested peptides were extracted. Tryptic peptide mixtures (1 μL) were applied on a ground steel MALDI plate with 1 μL of matrix solution, consisting of 0.8% CHCA (α-cyano-4-hydroxycinnamic acid, Sigma) in 50% ACN and 0.1% TFA.

2-D proteomic analysis and protein identification by mass spectrometry - HPLC-tandem MS/MS protein identification: A 10 μl aliquot of peptides were pre-concentrated at a flow of 5μl min-1 for 10 min using a C18 trap column (Acclaim PepMap RSLC, Thermo Scientific) and then loaded onto a 15 cm C18 column (75 μm ID, particle size 2 μm, 100Å, Acclaim PepMap RSLC, Thermo Scientific). The binary pumps of the HPLC (RSLC nano, Thermo Scientific) contained solution A (2% (v/v) ACN in 0.1% (v/v) formic acid) and solution B (80% ACN in 0.1% formic acid). The peptides were separated using a linear gradient of 4% - 40% B in 55 min at a flow rate of 300 nl/min. The column was placed in an oven operating at35 °C. Full scan MS spectra were acquired in the orbitrap (m/z 300–1600) in profile mode and data-dependent acquisition with the resolution set to 60,000 at m/z 400 and automatic gain control target at 10 6. The six most intense ions were sequentially isolated for collision-induced MS/MS fragmentation (normalized CID of 35%) and detection in the linear ion trap. Dynamic exclusion was set to 60 sec. Ions with single charge states were excluded. Lock mass of m/z445,120025 was used for internal calibration.

1.4. Transcription analysis - RT-q-PCR conditions

RT-q-PCR contained 5μl of 2x SYBR Green PCR MasterMix (Kapa, Finland), 20 pmoles of each primer, 1 μl of template cDNA and sterile distilled water to a final

volume of 10 µl. Thermal conditions were 95°C for 3 min followed by 40 cycles of 95°C for 15 sec and 20 sec at different annealing temperatures depending on the target gene (Supplementary Table S3 online). For detection of amplification artefacts a melting-curve analysis was performed immediately after completion of the RT-q-PCR run (95°C for 15 s, 55°C for 30 s, and slowly increasing to 95°C).

References

1. Parks, D., Imelfort, M., Skennerton, C., Hugenholtz, P. & Tyson, G. CheckM: assessing the quality of microbial genomes recovered from isolates, single cells, and metagenomes. Genome Res. doi:10.1101/gr.186072.114 (2015).
2. Seemann, T. Prokka: rapid prokaryotic genome annotation. Bioinformatics 30, 2068-2069 (2014).
3. Hyatt, D. *et al.* Prodigal: prokaryotic gene recognition and translation initiation site identification. *BMC Bioinf.* **11**, 119 (2010).
4. Laslett, D. & Canback, B. ARAGORN, a program to detect tRNA genes and tmRNA genes in nucleotide sequences. *Nucleic Acids Res.* **32**, 11-16 (2004).
5. Bland, C. *et al*. CRISPR Recognition Tool (CRT): a tool for automatic detection of clustered regularly interspaced palindromic repeats. *BMC Bioinf.* **8**, 209 (2007).
6. Camacho C, *et al.* BLAST+: architecture and applications. *BMC Bioinf.* **10**, 421 (2009).
7. Apweiler, R. *et al*. UniProt: the Universal Protein knowledgebase. *Nucleic Acids Res*. **32**, D115–D119 (2004).
8. Eddy, S. R. Accelerated profile HMM searches. *PLoS Comput. Biol*. **7**, e1002195 (2011).
9. Finn, R. D. *et al*. Pfam: the protein families database. *Nucleic Acids Res*. **42**, D222-D230 (2014).

10. Haft, D. H. *et al*. TIGRFAMs and Genome Properties in 2013. *Nucleic Acids Res*. **41**, D387-D395 (2013).

11. Li, W., Jaroszewski, L. & Godzik, A**.** Clustering of highly homologous sequences to reduce the size of large protein databases. *Bioinformatics* **17**, 282-283 (2001).

12. Edgar, R. C. MUSCLE: multiple sequence alignment with high accuracy and high throughput. *Nucleic Acids Res*. **32**, 1792-1797 (2004).

13. Talavera, G. & Castresana, J. Improvement of phylogenies after removing divergent and ambiguously aligned blocks from protein sequence alignments. *Syst. Biol*. **56,** 564-577 (2007).

14. Stamatakis, A. RAxML version 8: A tool for phylogenetic analysis and post-analysis of large phylogenies. *Bioinformatics* **30** (9), 1312-1313 (2014).

15. Abascal, F., Zardoya, R. & Posada, D**.** ProtTest: selection of best-fit models of protein evolution. *Bioinformatics* **21**, 2104-2105 (2005).

16. Paradis, E., Claude, J. & Strimmer, K. APE: Analyses of phylogenetics and evolution in R language. *Bioinformatics* **20,** 289-290 (2004).

17. Schliep, K. P. Phangorn: phylogenetic analysis in R. *Bioinformatics* **27**, 592-593 (2011).

18. R Core Team. R: A language and environment for statistical computing, reference index version 3.2.2. 2015, from http://www.r-project.org/ (2015)

Supplementary Tables

Supplementary Table S3. A list of proteins, categorized according to their general function,identified via proteomic analysis to be significantly *($p<0.05$)* up-regulated further validated by 2-fold change in volume abundancein *S. haloaromaticamans* cells grown on *ortho*-phenylphenol (OPP) and/or benzoic acid (BA) compared to cells grown on succinate (Succ). Asterisks next to spots intensity ratio values indicate the level of statistical significance (*, **, *** for p<0.05, p<0.01 and p<0.001 respectively) in differences in the spot intensity between OPP, BA and succinate

Spot No.	Proteins	Spots intensity ratio	
		BA/ Succ	OPP/ Succ
	Stress-related proteins		
134	Alkyl hydroperoxide reductase	5.52	15.3**
64	Chaperone protein DnaK -1	9.69***	9.05**
72	Chaperone protein DnaK -2	12.33**	10.47**
73	Chaperone protein DnaK -3	13.02**	25.61**
108	60 kDa chaperonin-1	5.75***	9.27***
109	60 kDa chaperonin -2	2.81***	4.41*
26	60 kDa chaperonin -3	6.49**	8.09**
193	10 kDA chaperonin	3.28*	4.63
203	Superoxide dismutase	4.46**	6.87**
208	Cold-shock DNA-binding domain-containing protein	6.65*	115.9***
	Transporters and membrane-related proteins		
181	TonB-dependent receptor-1	2.77**	2.82

102	TonB-dependent receptor-2	3.66**	0.29
187	Protein YceI	16.31**	10.23**
38	Pesticin receptor precursor	3.78**	70.67**
156	Flagellin domain-containing protein-1	9.59**	31.74***
179	Flagellin domain-containing protein-2	59.86***	275.2***
162	Flagellin domain-containing protein-3	2.07	4.53**
	Energy production and biosynthesis proteins		
16	ATP synthase subunit alpha-1 (EP)[a]	2.92*	4.12
17	ATP synthase subunit alpha-1 (EP)	4.33**	2.39
48	ATP synthase subunit beta-1 (EP)	3.35	14.74**
182	ATP synthase subunit beta-2 (EP)	3.5	7.37***
214	2,3-bisphosphoglycerate-dependent phosphoglycerate mutase (EP)	62.34***	29.19*
67	NADP-dependent malic enzyme (EP)	30.93**	25.18**
210	Putative NADH dehydrogenase/NAD(P)H nitroreductase (EP)	19.28**	29.58*
206	Dihydrolipoyl dehydrogenase (EP)	8.2***	7.13*
9	Transketolase (EP)	24.59***	20.36
217	Electron transfer flavoprotein subunit alpha/beta like protein (EP)	11.44**	31.49***
205	Ubiquinol-cytochrome c reductase iron-sulfur subunit (EP)	336.6***	27.71
225	Fructose-bisphosphate aldolase (EP)	7.95***	11.44**
82	Riboflavin biosynthesis protein RibBA (EP)	2.26**	4.54***
199	Aconitate hydratase-1 (EP)	5.35***	4.73*
56	Aconitate hydratase-2 (EP)	5.76**	8.89*
40	Aconitate hydratase-3 (EP)	2.94*	4.36*

224	Succinyl-CoA ligase [ADP-forming] alpha subunit (EP)	6.89***	12.79**
65	Nucleoside diphosphate kinase (EP)	9.01	37.87*
216	50S ribosomal protein L9-1 (PB)[a]	2.14**	1.95
189	50S ribosomal protein L9-2 (PB)	23.26***	32.78***
143	30S ribosomal protein S1-1 (PB)	17.02***	19.87**
115	30S ribosomal protein S1-2 (PB)	27.01**	15.84**
47	30S ribosomal protein S1-3 (PB)	79.77**	42.99*
184	Elongation factor P (PB)	3.88***	7.41**
196	Elongation factor Ts (PB)	2.9***	6.1***
71	Elongation factor Tu-1 (PB)	20.7**	53.75**
120	Elongation factor Tu-2 (PB)	12.51**	18.31***
126	Elongation factor Tu-3 (PB)	1.43	2.62*
150	Elongation factor G (PB)	7.98***	10.81**
74	DNA polymerase III subunit beta (DNA B)[a]	2.9**	4.47**
81	Aspartate Aminotransferase (AAB)[a]	21.04***	39.24**
92	Aspartate-semialdehyde dehydrogenase-1 (AAB)	3.4**	5.58**
98	Aspartate-semialdehyde dehydrogenase-2 (AAB)	3.38**	3.79**
96	Aspartate-semialdehyde dehydrogenase-3 (AAB)	1.31*	2.01**
163	D-3-phosphoglycerate dehydrogenase(AAB)	4.85**	0.81
230	Ketol-acid reductoisomerase (AAB)	6.8***	3.1*
4	Chorismate synthase (AAB)	3.25**	12.56**

[a] EP: Energy Production; PB: Protein Biosynthesis; DNA B: DNA biosynthesis; AAB: Amino Acids Biosynthesis

Supplementary Table S4. A list of the set of the primers used for the transcription analysis of the putative catabolic genes.

Target Gene	Gene Code		Primer sequence (5'-3')	Amplicon size(bp)	T_a (°C)
gyrB	BHE75_0369	f	GGCAGCTTGACCGAGACGAT	216	Var.[a]
		r	GGAATGGAATGACAGCTATTACGAG		
oppA1	BHE75_04573	f	ACATCAACAATCCGCCCGT	209	55
		r	GTTCGAACCCAGACCCTTCAGT		
oppA2	BHE75_04585	f	AGAGCCAGCTGGGTGATAGA	171	55
		r	CCGTCCCTGCCTGTATTC		
oppC	BHE75_04576	f	CAAGTGACGAACGAACCTGA	172	55
		r	CCTGCTCACCGGTAACAAAT		
oppD1	BHE75_04572	f	ACCGCTTTCTCGCCAATGTC	221	55
		r	AATCTTGAGCCCCTCTGTC		
oppD2	BHE75_04587	f	TGGTTCCCATGATCGCCTTGTAG	169	55
		r	GCGTTGTCGGCCATTCTAAACT		
bphH1	BHE75_04578	f	ATCGGCTGACACCACGGCA	154	55
		r	ATCGGACAGGACGAAGAACGC		
bphH2	BHE75_04595	f	CGAGTTGCGTCGTGCCTATC	183	55
		r	CCGAGTTGGCGTTGGACC		
bphI	BHE75_04580	f	CTGCAAGGCTCCAGCTTCAACTAC	169	55
		r	GCACCGAACGCACACCCAAC		
benA1	BHE75_01134	f	CGTCGTAGATGTAGGAGGAGTT	240	55
		r	AGGTGAAGGACGGCAAGAC		
benA2	BHE75_04555	f	CTCCGCCCGCTGGTCACTCT	195	55
		r	GGCGAATTGTGGAACGACCTCA		
benB1	BHE75_01133	f	GTCGACGGTGTAGAAGGAGGTG	183	55
		r	CGCATCAAGACGGAACGGTC		
benB2	BHE75_04554	f	GATCTTCCAGACCTTGCCTG	203	55
		r	GAGCGTGCTCATTGAAGTTACC		
benC2	BHE75_04560	f	CGGATTCGAGGCGGATCAC	250	58
		r	GAAGGCGATGGCGTCAAGG		
benD1	BHE75_01131	f	GTCACCTGCCGGACGACATC	271	65
		r	TGCCGGCGATGCTGGAAG		
benD2	BHE75_04553	f	GCCATCCACGCCTGTTCCTG	243	65

		r	GATGCTGGCGCAAGGTTCG		
catA1	BHE75_01135	f	TATTTCGCGCCGAGGTTGC	288	55
		r	CGTCATCACCGATGCCGAG		
catA2	BHE75_04556	f	GCTGGTGGTGTCATCGGGAT	159	55
		r	GAGCACTTCATGGACCTCTACA		
catB1	BHE75_01137	f	CGCGGCATCGGCTTTCAG	154	55
		r	CGTCTTCGTCCGCATCCACTG		
catB2	BHE75_04558	f	CGAACGCGGACCATTGAG	233	55
		r	CGCATGTGGGGGCAATCTG		
catC1	BHE75_01136	f	GGCAGCGTGCTCACCAGA	146	60
		r	AAGCGCGTGGAACGGGAA		
catC2	BHE75_04557	f	GGCAGGCCGATAAGGATGT	179	60
		r	CTCGGCTTCGATGCGGAC		
pcaD1	BHE75_01127	f	CATGCCGCCCTTGGACAATC	189	60
		r	TGGACGCCGCAGATGGATG		
pcaD2	BHE75_04550	f	ATGGCGTAGTCCTCGTCTGC	188	60
		r	GGAAAGCGCGTCTATTGGAAG		
pcaI1	BHE75_01130	f	CCGACAGATACTGGCTTTC	271	55
		r	GACAAAATCTATCCGTCGC		
pcaI2	BHE75_04552	f	CTTCACTTGGCGGCTCTTCA	162	55
		r	GGGATGACCGTGATGTCTG		
pcaF1	BHE75_01128	f	GCTTGAGGCCGTGACGCTGC	221	60
		r	AATCGTTCCCGTCATAATCGCCG		
pcaF2	BHE75_04549	f	AAGTGTAGTGTCCTCGATCTTCTGG	310	60
		r	GGACCTCGATTGGGCACAG		
bphA1	BHE75_04590	f	GATGACGCCCGAGGATGTTC	243	60
		r	AAGACCGTCATGTGCGAGCC		
bphA2	BHE75_04591	f	TTGATCTTCTTGCCGATGACATTC	209	60
		r	GTTGCTGACCAGATGCCGTGT		
bphA3	BHE75_04592	f	GCATGAGGAGCCGGTAGCACT	214	60
		r	CTCGATGCGCACCGGATAGAGC		
bphA4	BHE75_04593	f	GGCTTGAGCGTCACGATAGTC	221	60
		r	GCACCGATGCTGACGAGAAC		
benR1	BHE75_01138	f	CCGATGGCGCTCACCGATG	159	60
		r	CAGGCGGGCGTAGAGAACC		
benR2	BHE75_04559	f	CCATGATGAGGACCGCTGC	242	60

		r	CAGAATGGTCCGCTTGATGTCT		
oppR	BHE75_04570	f	GCCTTGAGAGTTCAGTGCGTCTA	177	60
		r	GCAGTTATCATGTGCCAGCGAG		
bphR1	BHE75_04588	f	GCTCGAGATCCATATTGCCG	258	60
		r	ATCTCGTCGCCGTCAGGTAG		
bphR2	BHE75_04589	f	GTCCCGATGACCAGCCAAC	215	60
		r	ATATCCGATCTCCATCCAGCAG		

[a]The T_a of *gyrB* varied from 55 to 65°C depending on the gene amplified in parallel

Supplementary Figures

(a)

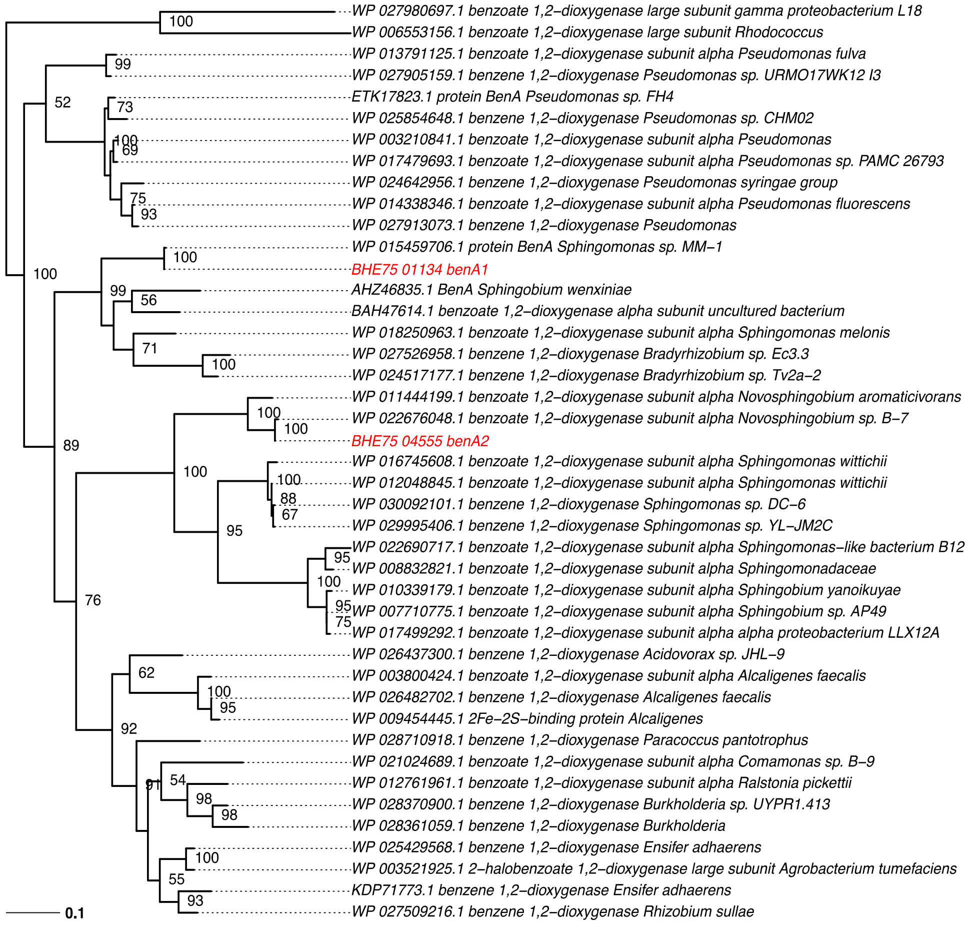

(b)

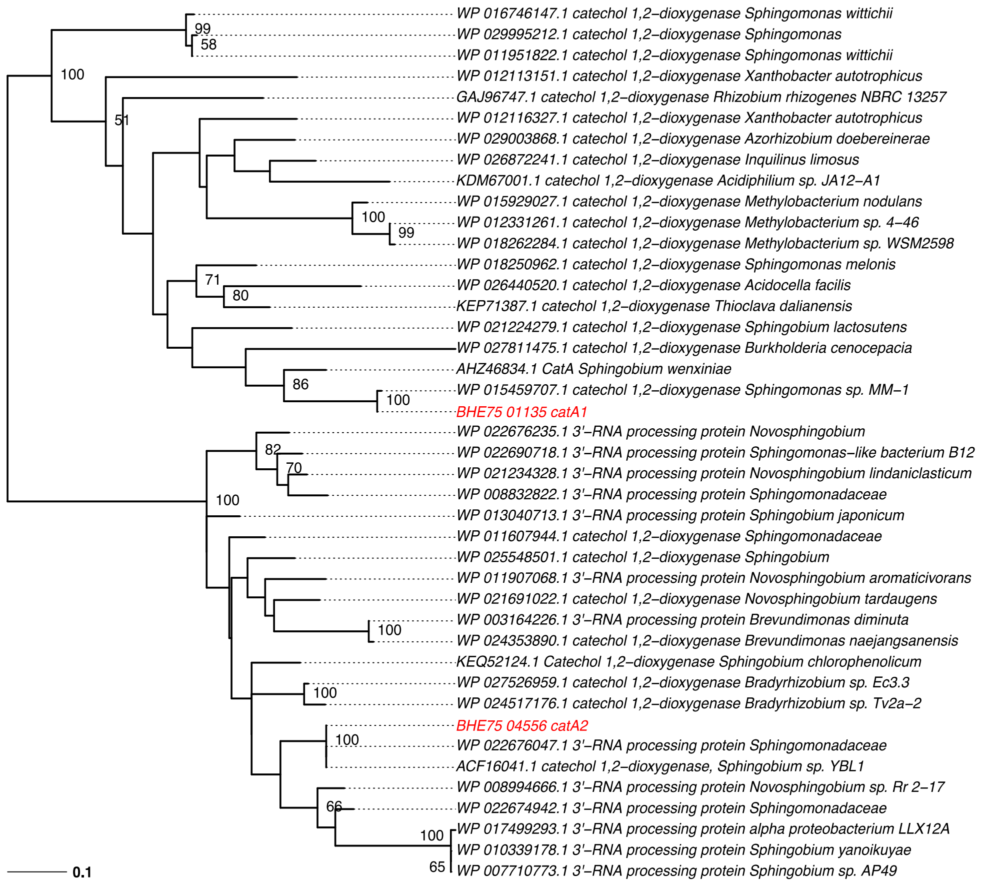
WP 016746147.1 catechol 1,2–dioxygenase Sphingomonas wittichii
WP 029995212.1 catechol 1,2–dioxygenase Sphingomonas
WP 011951822.1 catechol 1,2–dioxygenase Sphingomonas wittichii
WP 012113151.1 catechol 1,2–dioxygenase Xanthobacter autotrophicus
GAJ96747.1 catechol 1,2–dioxygenase Rhizobium rhizogenes NBRC 13257
WP 012116327.1 catechol 1,2–dioxygenase Xanthobacter autotrophicus
WP 029003868.1 catechol 1,2–dioxygenase Azorhizobium doebereinerae
WP 026872241.1 catechol 1,2–dioxygenase Inquilinus limosus
KDM67001.1 catechol 1,2–dioxygenase Acidiphilium sp. JA12–A1
WP 015929027.1 catechol 1,2–dioxygenase Methylobacterium nodulans
WP 012331261.1 catechol 1,2–dioxygenase Methylobacterium sp. 4–46
WP 018262284.1 catechol 1,2–dioxygenase Methylobacterium sp. WSM2598
WP 018250962.1 catechol 1,2–dioxygenase Sphingomonas melonis
WP 026440520.1 catechol 1,2–dioxygenase Acidocella facilis
KEP71387.1 catechol 1,2–dioxygenase Thioclava dalianensis
WP 021224279.1 catechol 1,2–dioxygenase Sphingobium lactosutens
WP 027811475.1 catechol 1,2–dioxygenase Burkholderia cenocepacia
AHZ46834.1 CatA Sphingobium wenxiniae
WP 015459707.1 catechol 1,2–dioxygenase Sphingomonas sp. MM–1
BHE75 01135 catA1
WP 022676235.1 3'–RNA processing protein Novosphingobium
WP 022690718.1 3'–RNA processing protein Sphingomonas–like bacterium B12
WP 021234328.1 3'–RNA processing protein Novosphingobium lindaniclasticum
WP 008832822.1 3'–RNA processing protein Sphingomonadaceae
WP 013040713.1 3'–RNA processing protein Sphingobium japonicum
WP 011607944.1 catechol 1,2–dioxygenase Sphingomonadaceae
WP 025548501.1 catechol 1,2–dioxygenase Sphingobium
WP 011907068.1 3'–RNA processing protein Novosphingobium aromaticivorans
WP 021691022.1 catechol 1,2–dioxygenase Novosphingobium tardaugens
WP 003164226.1 3'–RNA processing protein Brevundimonas diminuta
WP 024353890.1 catechol 1,2–dioxygenase Brevundimonas naejangsanensis
KEQ52124.1 Catechol 1,2–dioxygenase Sphingobium chlorophenolicum
WP 027526959.1 catechol 1,2–dioxygenase Bradyrhizobium sp. Ec3.3
WP 024517176.1 catechol 1,2–dioxygenase Bradyrhizobium sp. Tv2a–2
BHE75 04556 catA2
WP 022676047.1 3'–RNA processing protein Sphingomonadaceae
ACF16041.1 catechol 1,2–dioxygenase, Sphingobium sp. YBL1
WP 008994666.1 3'–RNA processing protein Novosphingobium sp. Rr 2–17
WP 022674942.1 3'–RNA processing protein Sphingomonadaceae
WP 017499293.1 3'–RNA processing protein alpha proteobacterium LLX12A
WP 010339178.1 3'–RNA processing protein Sphingobium yanoikuyae
WP 007710773.1 3'–RNA processing protein Sphingobium sp. AP49
0.1

(c)

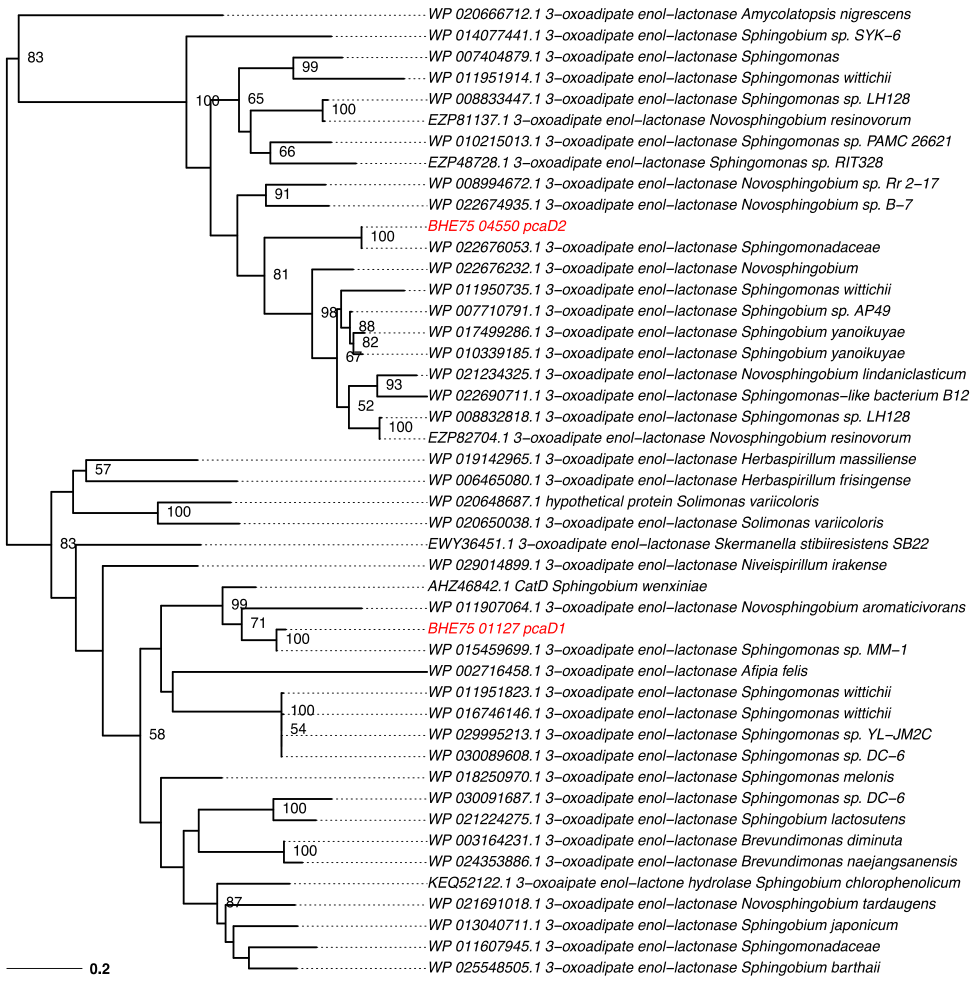

Supplementary Figure S1. Phylogenetic analysis of the translated products of genes (a) *benA1/benA2,* (b) *catA1 /catA2,* (c) *pcaD1/pcaD2* localized in the orthologous operons 1 and 2 of the genome of the *Sphingomonas haloaromaticamans* strain P3

(a)

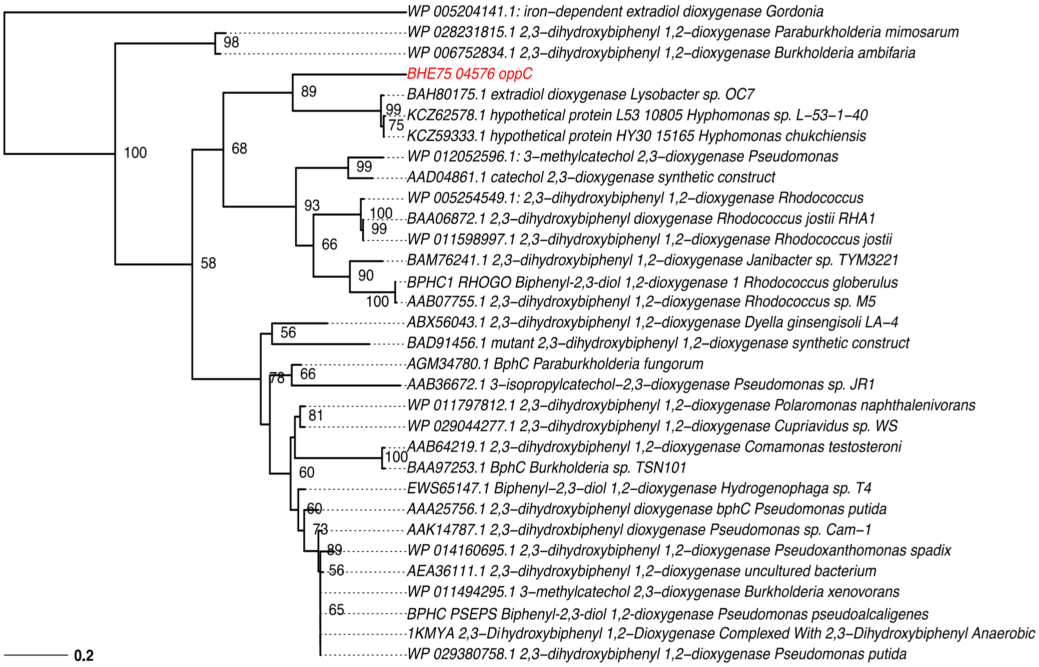
WP 005204141.1: iron–dependent extradiol dioxygenase Gordonia
WP 028231815.1 2,3–dihydroxybiphenyl 1,2–dioxygenase Paraburkholderia mimosarum
WP 006752834.1 2,3–dihydroxybiphenyl 1,2–dioxygenase Burkholderia ambifaria
BHE75 04576 oppC
BAH80175.1 extradiol dioxygenase Lysobacter sp. OC7
KCZ62578.1 hypothetical protein L53 10805 Hyphomonas sp. L–53–1–40
KCZ59333.1 hypothetical protein HY30 15165 Hyphomonas chukchiensis
WP 012052596.1: 3–methylcatechol 2,3–dioxygenase Pseudomonas
AAD04861.1 catechol 2,3–dioxygenase synthetic construct
WP 005254549.1: 2,3–dihydroxybiphenyl 1,2–dioxygenase Rhodococcus
BAA06872.1 2,3–dihydroxybiphenyl dioxygenase Rhodococcus jostii RHA1
WP 011598997.1 2,3–dihydroxybiphenyl 1,2–dioxygenase Rhodococcus jostii
BAM76241.1 2,3–dihydroxybiphenyl 1,2–dioxygenase Janibacter sp. TYM3221
BPHC1 RHOGO Biphenyl-2,3-diol 1,2-dioxygenase 1 Rhodococcus globerulus
AAB07755.1 2,3–dihydroxybiphenyl 1,2–dioxygenase Rhodococcus sp. M5
ABX56043.1 2,3–dihydroxybiphenyl 1,2–dioxygenase Dyella ginsengisoli LA–4
BAD91456.1 mutant 2,3–dihydroxybiphenyl 1,2–dioxygenase synthetic construct
AGM34780.1 BphC Paraburkholderia fungorum
AAB36672.1 3–isopropylcatechol–2,3–dioxygenase Pseudomonas sp. JR1
WP 011797812.1 2,3–dihydroxybiphenyl 1,2–dioxygenase Polaromonas naphthalenivorans
WP 029044277.1 2,3–dihydroxybiphenyl 1,2–dioxygenase Cupriavidus sp. WS
AAB64219.1 2,3–dihydroxybiphenyl 1,2–dioxygenase Comamonas testosteroni
BAA97253.1 BphC Burkholderia sp. TSN101
EWS65147.1 Biphenyl–2,3–diol 1,2–dioxygenase Hydrogenophaga sp. T4
AAA25756.1 2,3–dihydroxybiphenyl dioxygenase bphC Pseudomonas putida
AAK14787.1 2,3–dihydroxbiphenyl dioxygenase Pseudomonas sp. Cam–1
WP 014160695.1 2,3–dihydroxybiphenyl 1,2–dioxygenase Pseudoxanthomonas spadix
AEA36111.1 2,3–dihydroxybiphenyl 1,2–dioxygenase uncultured bacterium
WP 011494295.1 3–methylcatechol 2,3–dioxygenase Burkholderia xenovorans
BPHC PSEPS Biphenyl-2,3-diol 1,2-dioxygenase Pseudomonas pseudoalcaligenes
1KMYA 2,3–Dihydroxybiphenyl 1,2–Dioxygenase Complexed With 2,3–Dihydroxybiphenyl Anaerobic
WP 029380758.1 2,3–dihydroxybiphenyl 1,2–dioxygenase Pseudomonas putida
0.2

(b)

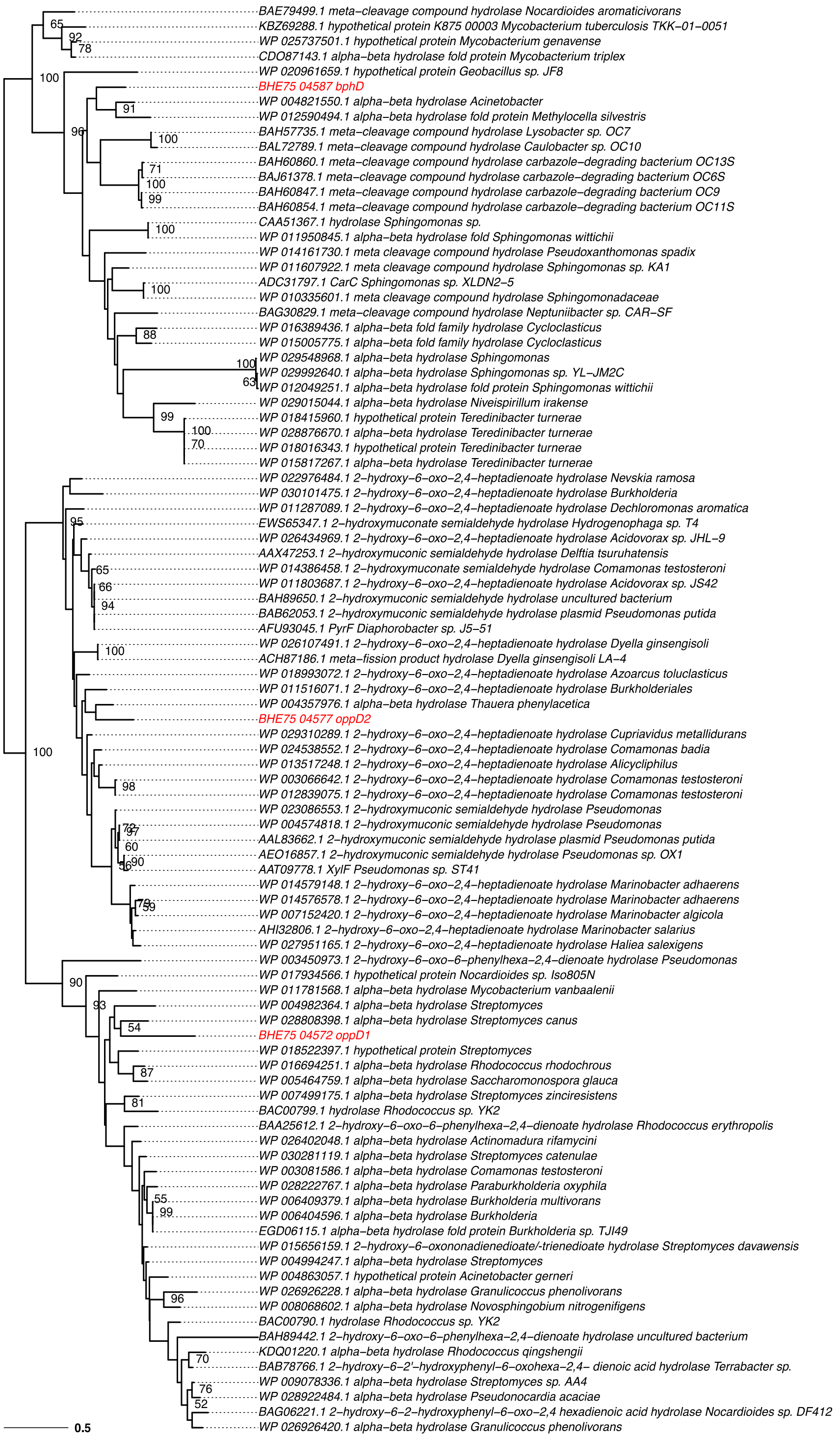
BAE79499.1 meta–cleavage compound hydrolase Nocardioides aromaticivorans
KBZ69288.1 hypothetical protein K875 00003 Mycobacterium tuberculosis TKK–01–0051
WP 025737501.1 hypothetical protein Mycobacterium genavense
CDO87143.1 alpha–beta hydrolase fold protein Mycobacterium triplex
WP 020961659.1 hypothetical protein Geobacillus sp. JF8
BHE75 04587 bphD
WP 004821550.1 alpha–beta hydrolase Acinetobacter
WP 012590494.1 alpha–beta hydrolase fold protein Methylocella silvestris
BAH57735.1 meta–cleavage compound hydrolase Lysobacter sp. OC7
BAL72789.1 meta–cleavage compound hydrolase Caulobacter sp. OC10
BAH60860.1 meta–cleavage compound hydrolase carbazole–degrading bacterium OC13S
BAJ61378.1 meta–cleavage compound hydrolase carbazole–degrading bacterium OC6S
BAH60847.1 meta–cleavage compound hydrolase carbazole–degrading bacterium OC9
BAH60854.1 meta–cleavage compound hydrolase carbazole–degrading bacterium OC11S
CAA51367.1 hydrolase Sphingomonas sp.
WP 011950845.1 alpha–beta hydrolase fold Sphingomonas wittichii
WP 014161730.1 meta cleavage compound hydrolase Pseudoxanthomonas spadix
WP 011607922.1 meta cleavage compound hydrolase Sphingomonas sp. KA1
ADC31797.1 CarC Sphingomonas sp. XLDN2–5
WP 010335601.1 meta cleavage compound hydrolase Sphingomonadaceae
BAG30829.1 meta–cleavage compound hydrolase Neptuniibacter sp. CAR–SF
WP 016389436.1 alpha–beta fold family hydrolase Cycloclasticus
WP 015005775.1 alpha–beta fold family hydrolase Cycloclasticus
WP 029548968.1 alpha–beta hydrolase Sphingomonas
WP 029992640.1 alpha–beta hydrolase Sphingomonas sp. YL–JM2C
WP 012049251.1 alpha–beta hydrolase fold protein Sphingomonas wittichii
WP 029015044.1 alpha–beta hydrolase Niveispirillum irakense
WP 018415960.1 hypothetical protein Teredinibacter turnerae
WP 028876670.1 alpha–beta hydrolase Teredinibacter turnerae
WP 018016343.1 hypothetical protein Teredinibacter turnerae
WP 015817267.1 alpha–beta hydrolase Teredinibacter turnerae
WP 022976484.1 2–hydroxy–6–oxo–2,4–heptadienoate hydrolase Nevskia ramosa
WP 030101475.1 2–hydroxy–6–oxo–2,4–heptadienoate hydrolase Burkholderia
WP 011287089.1 2–hydroxy–6–oxo–2,4–heptadienoate hydrolase Dechloromonas aromatica
EWS65347.1 2–hydroxymuconate semialdehyde hydrolase Hydrogenophaga sp. T4
WP 026434969.1 2–hydroxy–6–oxo–2,4–heptadienoate hydrolase Acidovorax sp. JHL–9
AAX47253.1 2–hydroxymuconic semialdehyde hydrolase Delftia tsuruhatensis
WP 014386458.1 2–hydroxymuconate semialdehyde hydrolase Comamonas testosteroni
WP 011803687.1 2–hydroxy–6–oxo–2,4–heptadienoate hydrolase Acidovorax sp. JS42
BAH89650.1 2–hydroxymuconic semialdehyde hydrolase uncultured bacterium
BAB62053.1 2–hydroxymuconic semialdehyde hydrolase plasmid Pseudomonas putida
AFU93045.1 PyrF Diaphorobacter sp. J5–51
WP 026107491.1 2–hydroxy–6–oxo–2,4–heptadienoate hydrolase Dyella ginsengisoli
ACH87186.1 meta–fission product hydrolase Dyella ginsengisoli LA–4
WP 018993072.1 2–hydroxy–6–oxo–2,4–heptadienoate hydrolase Azoarcus toluclasticus
WP 011516071.1 2–hydroxy–6–oxo–2,4–heptadienoate hydrolase Burkholderiales
WP 004357976.1 alpha–beta hydrolase Thauera phenylacetica
BHE75 04577 oppD2
WP 029310289.1 2–hydroxy–6–oxo–2,4–heptadienoate hydrolase Cupriavidus metallidurans
WP 024538552.1 2–hydroxy–6–oxo–2,4–heptadienoate hydrolase Comamonas badia
WP 013517248.1 2–hydroxy–6–oxo–2,4–heptadienoate hydrolase Alicycliphilus
WP 003066642.1 2–hydroxy–6–oxo–2,4–heptadienoate hydrolase Comamonas testosteroni
WP 012839075.1 2–hydroxy–6–oxo–2,4–heptadienoate hydrolase Comamonas testosteroni
WP 023086553.1 2–hydroxymuconic semialdehyde hydrolase Pseudomonas
WP 004574818.1 2–hydroxymuconic semialdehyde hydrolase Pseudomonas
AAL83662.1 2–hydroxymuconic semialdehyde hydrolase plasmid Pseudomonas putida
AEO16857.1 2–hydroxymuconic semialdehyde hydrolase Pseudomonas sp. OX1
AAT09778.1 XylF Pseudomonas sp. ST41
WP 014579148.1 2–hydroxy–6–oxo–2,4–heptadienoate hydrolase Marinobacter adhaerens
WP 014576578.1 2–hydroxy–6–oxo–2,4–heptadienoate hydrolase Marinobacter adhaerens
WP 007152420.1 2–hydroxy–6–oxo–2,4–heptadienoate hydrolase Marinobacter algicola
AHI32806.1 2–hydroxy–6–oxo–2,4–heptadienoate hydrolase Marinobacter salarius
WP 027951165.1 2–hydroxy–6–oxo–2,4–heptadienoate hydrolase Haliea salexigens
WP 003450973.1 2–hydroxy–6–oxo–6–phenylhexa–2,4–dienoate hydrolase Pseudomonas
WP 017934566.1 hypothetical protein Nocardioides sp. Iso805N
WP 011781568.1 alpha–beta hydrolase Mycobacterium vanbaalenii
WP 004982364.1 alpha–beta hydrolase Streptomyces
WP 028808398.1 alpha–beta hydrolase Streptomyces canus
BHE75 04572 oppD1
WP 018522397.1 hypothetical protein Streptomyces
WP 016694251.1 alpha–beta hydrolase Rhodococcus rhodochrous
WP 005464759.1 alpha–beta hydrolase Saccharomonospora glauca
WP 007499175.1 alpha–beta hydrolase Streptomyces zincirestistens
BAC00799.1 hydrolase Rhodococcus sp. YK2
BAA25612.1 2–hydroxy–6–oxo–6–phenylhexa–2,4–dienoate hydrolase Rhodococcus erythropolis
WP 026402048.1 alpha–beta hydrolase Actinomadura rifamycini
WP 030281119.1 alpha–beta hydrolase Streptomyces catenulae
WP 003081586.1 alpha–beta hydrolase Comamonas testosteroni
WP 028222767.1 alpha–beta hydrolase Paraburkholderia oxyphila
WP 006409379.1 alpha–beta hydrolase Burkholderia multivorans
WP 006404596.1 alpha–beta hydrolase Burkholderia
EGD06115.1 alpha–beta hydrolase fold protein Burkholderia sp. TJI49
WP 015656159.1 2–hydroxy–6–oxononadienedioate/–trienedioate hydrolase Streptomyces davawensis
WP 004994247.1 alpha–beta hydrolase Streptomyces
WP 004863057.1 hypothetical protein Acinetobacter gerneri
WP 026926228.1 alpha–beta hydrolase Granulicoccus phenolivorans
WP 008068602.1 alpha–beta hydrolase Novosphingobium nitrogenifigens
BAC00790.1 hydrolase Rhodococcus sp. YK2
BAH89442.1 2–hydroxy–6–oxo–6–phenylhexa–2,4–dienoate hydrolase uncultured bacterium
KDQ01220.1 alpha–beta hydrolase Rhodococcus qingshengii
BAB78766.1 2–hydroxy–6–2′–hydroxyphenyl–6–oxohexa–2,4– dienoic acid hydrolase Terrabacter sp.
WP 009078336.1 alpha–beta hydrolase Streptomyces sp. AA4
WP 028922484.1 alpha–beta hydrolase Pseudonocardia acaciae
BAG06221.1 2–hydroxy–6–2–hydroxyphenyl–6–oxo–2,4 hexadienoic acid hydrolase Nocardioides sp. DF412
WP 026926420.1 alpha–beta hydrolase Granulicoccus phenolivorans
0.5

(c)

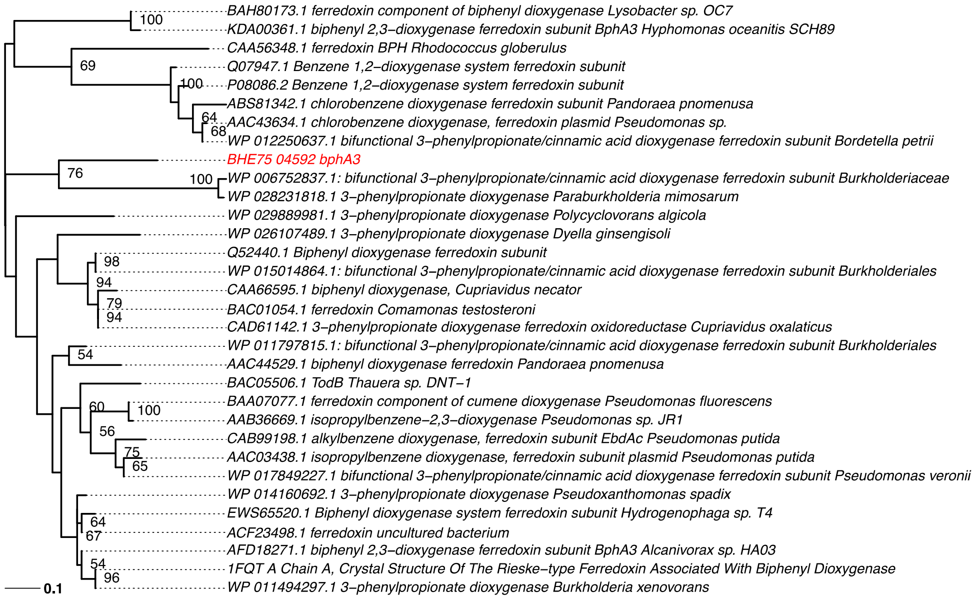

(d)

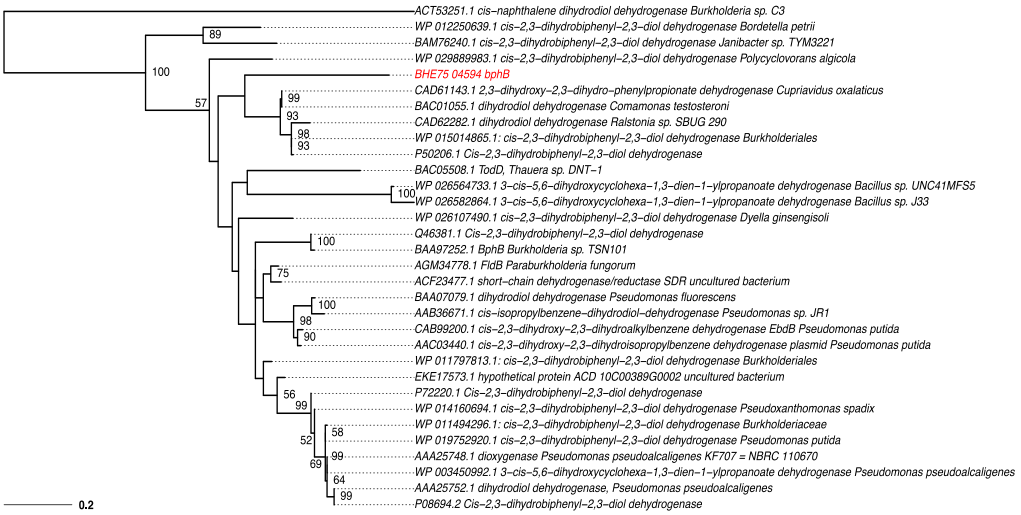

Supplementary Figure S2. Phylogenetic analysis of the translated products of the genes (a) *oppC*, (b) *oppD1/oppD2/bphD* (orthologs), (c) *bphA3* and (d) *bphB* localized in operons 3 and 4 of the genome of the *Sphingomonas haloaromaticamans* strain P3.

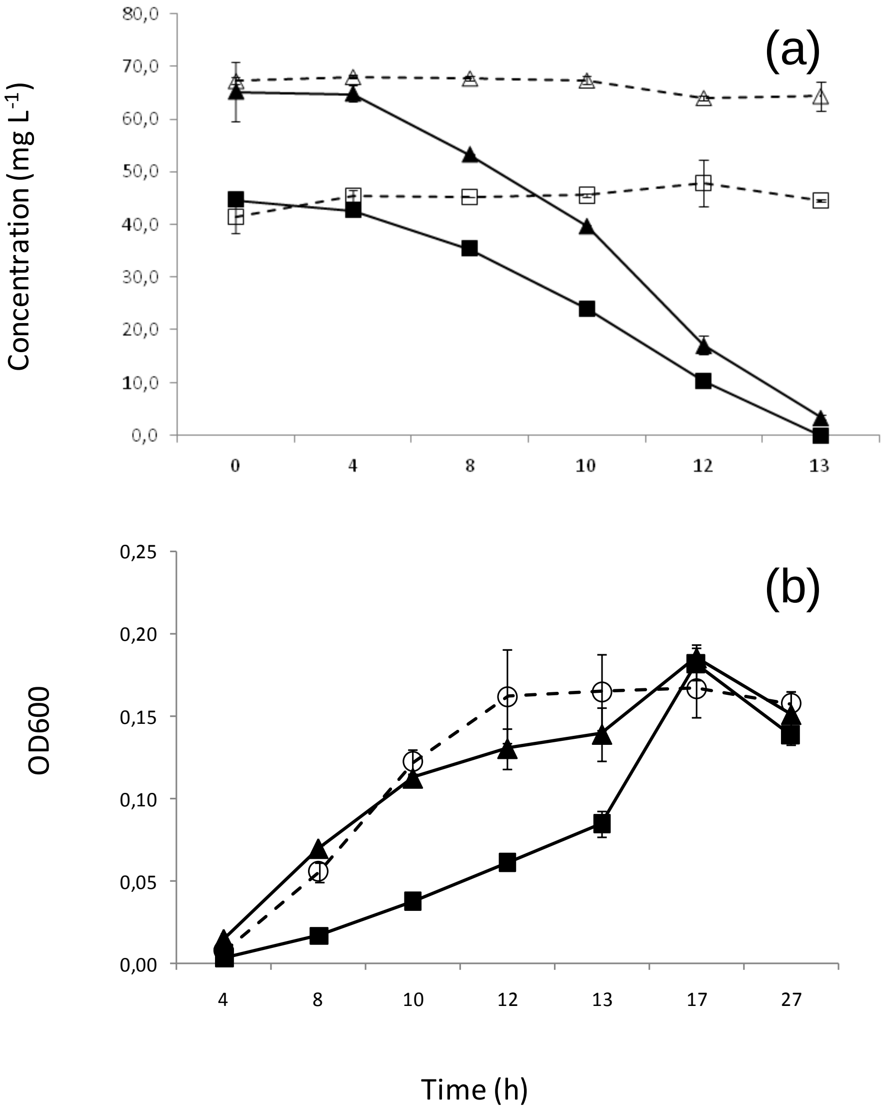

Supplementary Figure S3: (a) The degradation of ortho-phenylphenol (OPP) (■) and benzoic acid (BA) (▲) in MSMN + CA inoculated with the *Sphingomonas haloaromaticamans* strain P3 and used for the proteomic analysis. The degradation in corresponding non inoculated controls is also shown (open symbols. dotted lines); (b) the growth of *S. haloaromaticamans* in MSMN + CA + OPP (■). + BA (▲) or + succinate (○) as determined by OD_{600}. Each value is the mean of three replicates ± the standard deviation.

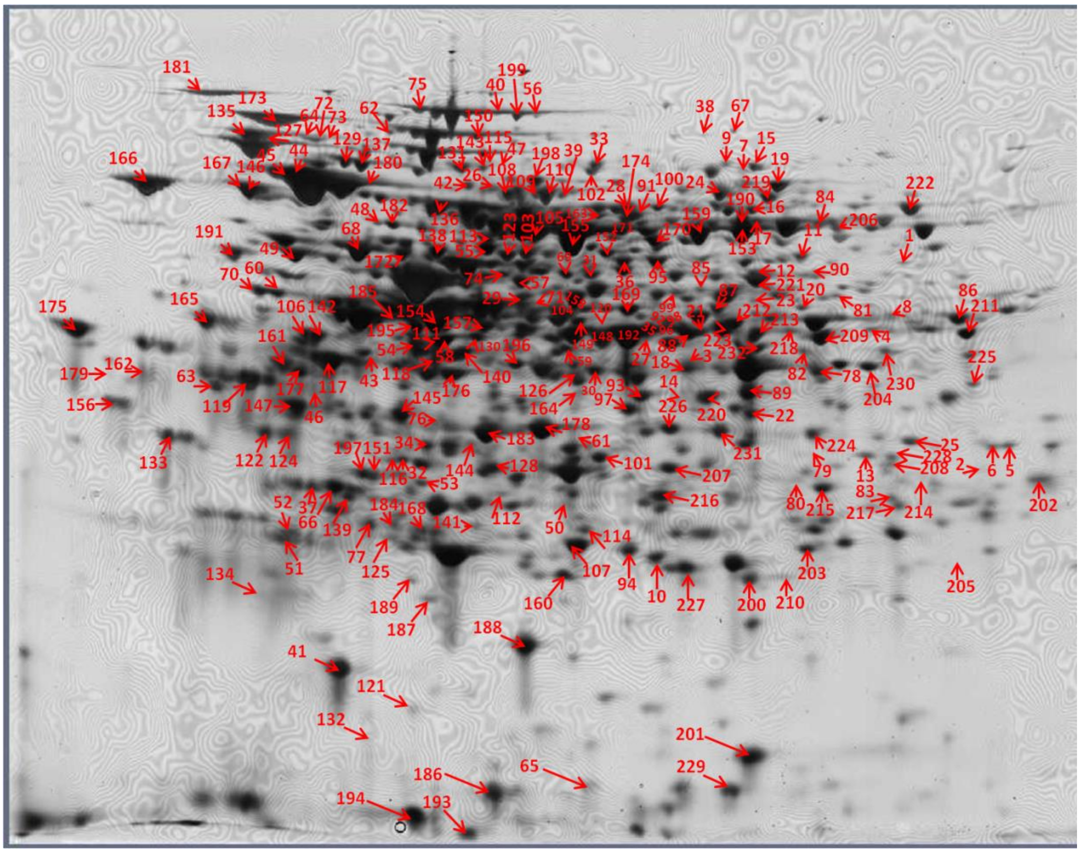

Supplementary Figure S4. Representative silver stained 2-DE-PAGE reference map of *Sphingomonas haloaromaticamans* grown in MSMN+CA+Succinate. Numbers correspond to the proteins listed in Supplementary Tables S1 and S2.

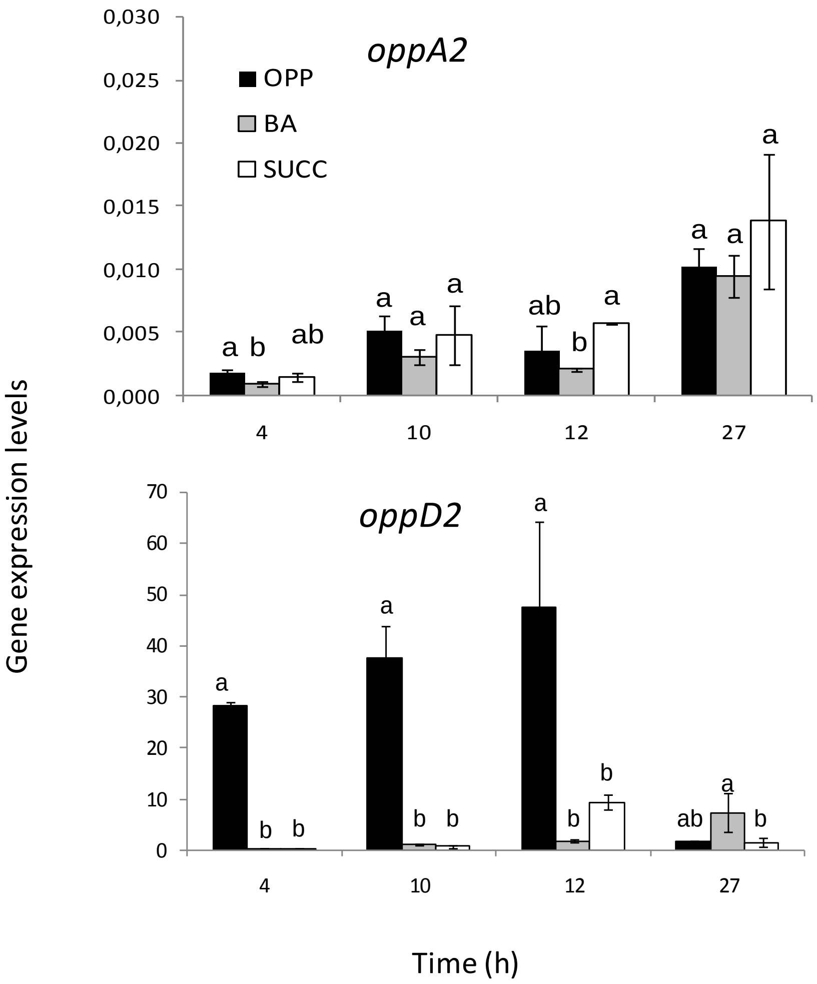

Supplementary Figure S5. The transcription patterns of genes *oppA2* and *oppD2* in cells of the *Sphingomonas haloaromaticamans* strain P3 growing in MSMN + CA + *ortho*-phenylphenol (OPP), benzoic acid (BA) or succinate (SUCC). These genes were localized in catabolic operons 3 and 4 and have a putative role in the upper OPP pathway. Each value is the mean of three replicates ± the standard deviation. Within each time point bars designated by the same letter are not significantly different at the 5% level.

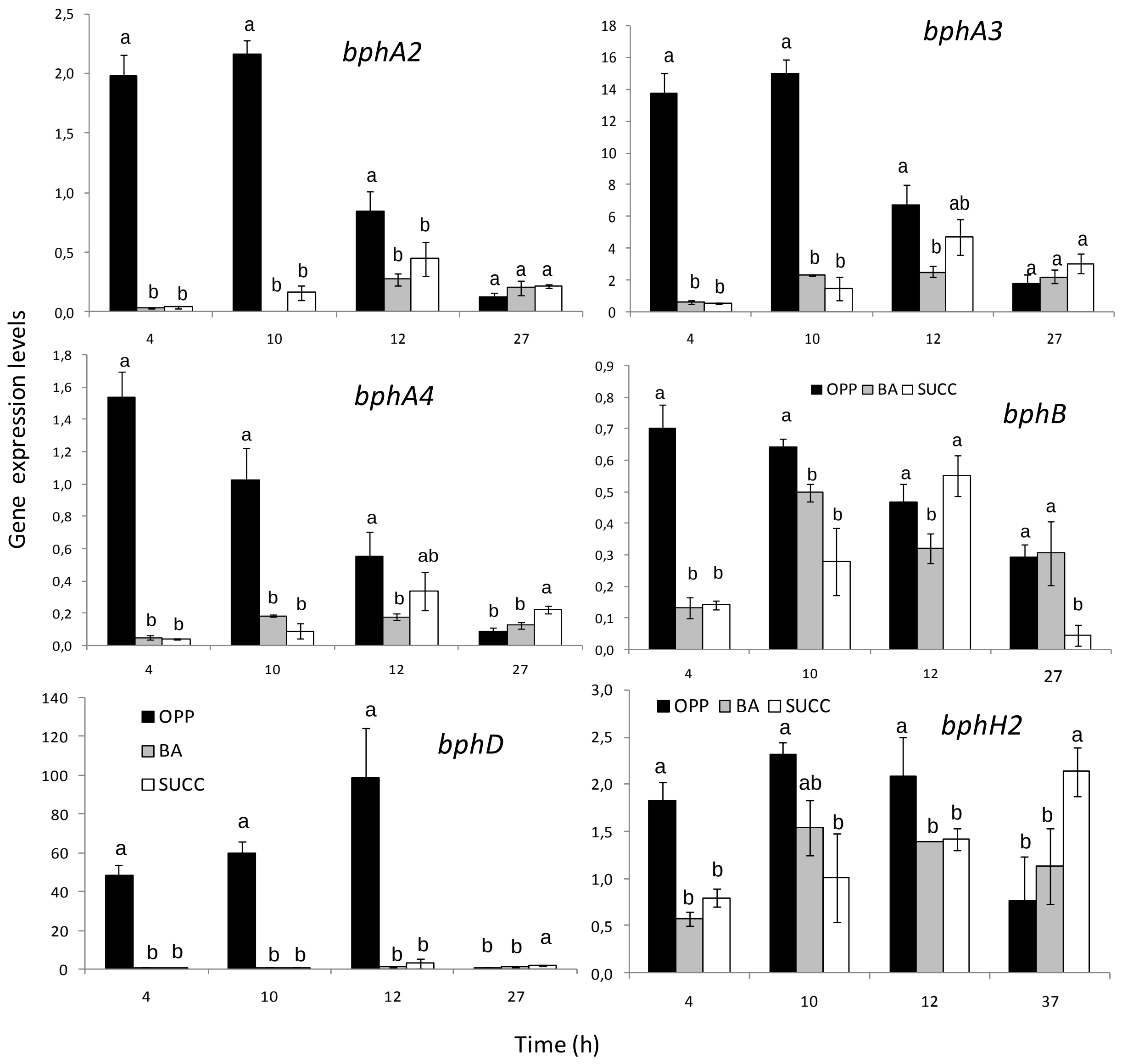

Supplementary Figure S6. The transcription patterns of genes b*phA2, bphA3, bphA4, bphB* , *bphD* and *bphH2* in cells of the *Sphingomonas haloaromaticamans* strain P3 growing on MSMN + CA + ortho-phenylphenol (OPP), benzoic acid (BA) or succinate (SUCC). All genes were localized in the bph operon 4 and had a putative role in the biphenyl pathway. Each value is the mean of three replicates + the standard deviation. At each time point bars designated by the same letter are not significantly different at the 5% level.

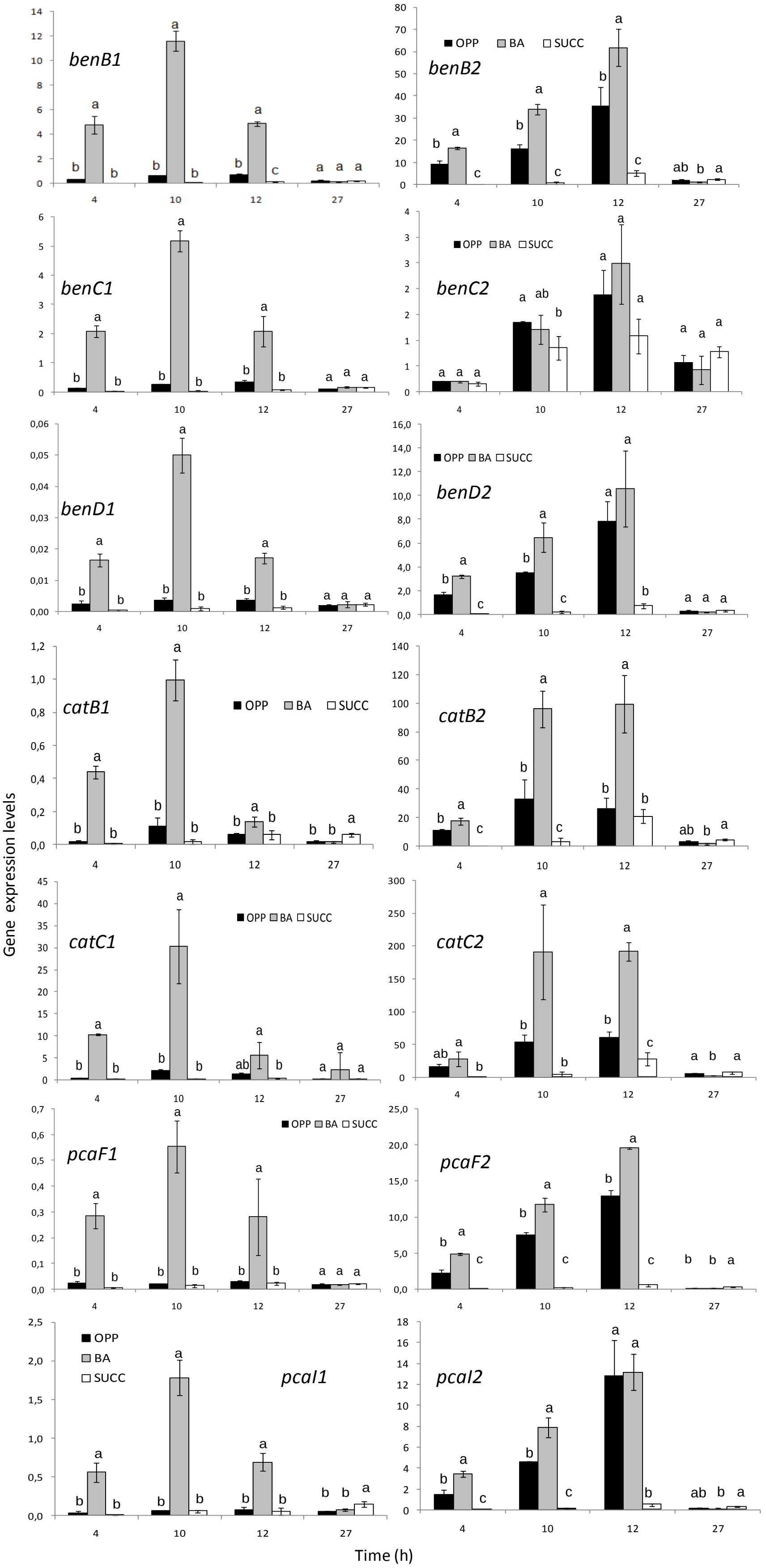

benB1
benB2
benC1
benC2
benD1
benD2
catB1
catB2
catC1
catC2
pcaF1
pcaF2
pcaI1
pcaI2
OPP
BA
SUCC
Gene expression levels
Time (h)
4
10
12
27

Supplementary Figure S7. The transcription patterns of genes *benB1/benB2*, *benC1/benC2 benD1/benD2*, *catB1/B2*, *catC1/C2*, *pca F1/pcaF2* and *pcaI1/pcaI2* in the cells of the *Sphingomonas haloaromaticamans* strain P3 growing in MSMN +CA + *ortho*-phenylphenol (OPP), benzoic acid (BA) or succinate (SUCC). These genes are localized in catabolic operons 1 and 2 and are involved in the transformation of BA (produced during degradation of OPP) via the *ortho* cleavage pathway. Each value is the mean of three replicates + the standard deviation. Within each time point bars designated by the same letter are not significantly different at the 5% level.

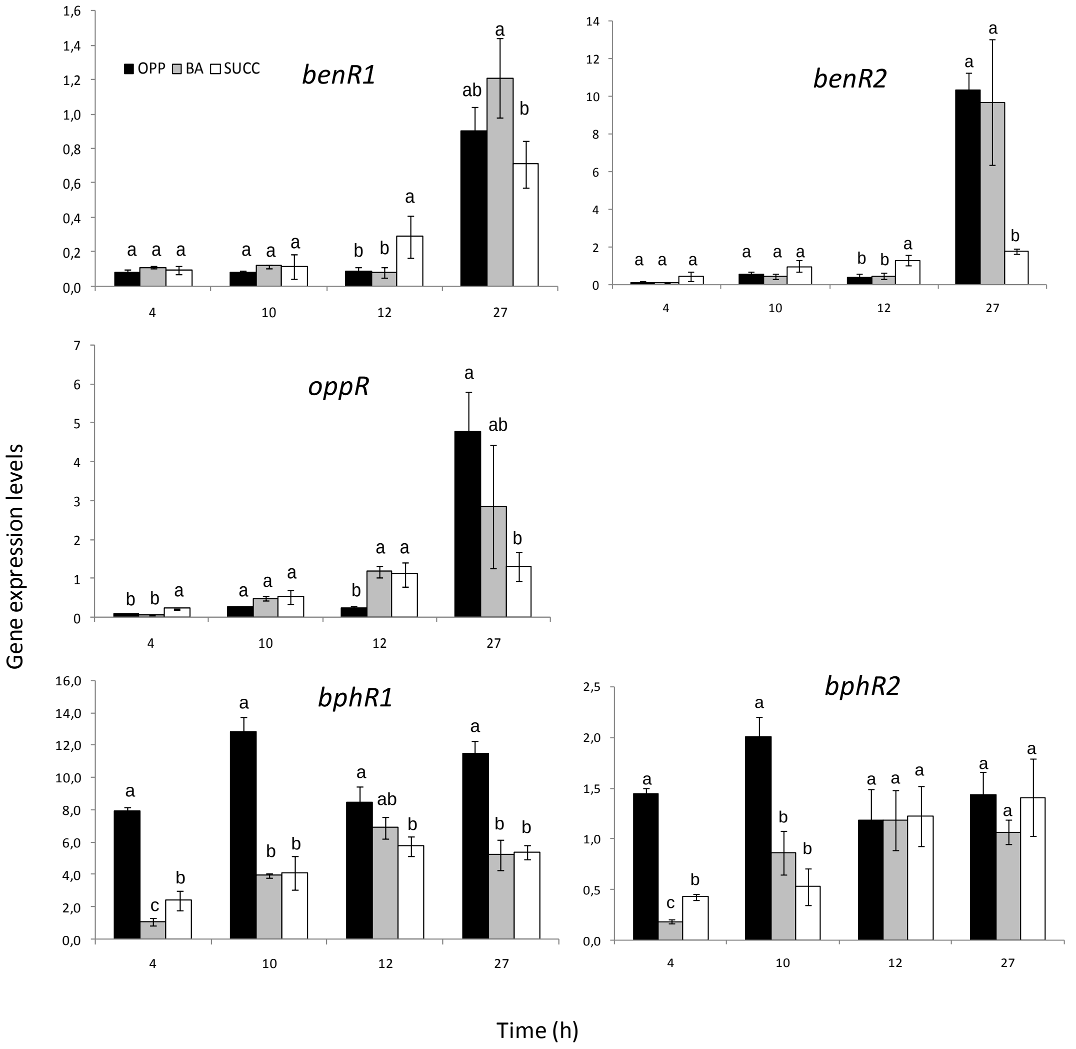

Supplementary Figure S8. The transcription patterns of transcriptional regulatory genes *benR1* & *benR2* (operons 1 and 2), *oppR* (operon 3) and *bphR1, bphR2* (operon 4) in cells of the *Sphingomonas haloaromaticamans* strain P3 growing on MSMN + CA + *ortho*-phenylphenol (OPP), benzoic acid (BA) or succinate (SUCC). Each value is the mean of three replicates ± the standard deviation. At each time point bars designated by the same letter are not significantly different at the 5% level.
